# PRISM: a clinically interpretable stepwise framework for multimodal skin cancer diagnosis

**DOI:** 10.1038/s41598-026-47756-4

**Published:** 2026-05-21

**Authors:** Pedro H. G. Bouzon, Wyctor F. da Rocha, Luis A. de Souza, André G. C. Pacheco

**Affiliations:** Federal University of Espìrito Santo,Graduate Program in Informatics, Vitória, Brazil

**Keywords:** Deep learning, Skin cancer classification, Explainable artificial intelligence, Multimodal methods, Cancer, Computational biology and bioinformatics, Health care, Mathematics and computing, Medical research

## Abstract

Skin cancer is the most common type of cancer worldwide, placing a substantial burden on healthcare systems. While numerous computer-assisted diagnostic systems have been developed, most offer limited transparency, making it difficult for healthcare professionals to understand how individual pieces of clinical information influence the diagnostic outcome. In this work, we propose PRISM: Probabilistic Reasoning Interpretable Stepwise Model, an interpretable multimodal skin cancer classification framework that integrates image-based predictions with clinical data using a Bayesian framework. Our approach allows the model to be evaluated with incrementally available metadata, enabling clinicians to interpret how each clinical feature contributes to the diagnostic decision. To address the compounding overconfidence inherent in sequential Bayesian updating, we implement a Stepwise Calibration protocol that dynamically scales with the volume of available evidence, ensuring statistically reliable confidence estimates at every intermediate diagnostic step. We validated the framework with four competitive vision backbones on three datasets. Compared to state-of-the-art attention-based methods, our framework achieves competitive or superior performance, reaching a peak balanced accuracy of $\mathbf {77.2 \pm 3.6\%}$ on the PAD-UFES-20 dataset. Finally, qualitative case studies indicate that PRISM creates a reasoning process that is consistent with clinical logic, suggesting it is a viable alternative for clinical decision support. An interactive demo is available at https://huggingface.co/spaces/pedrobouzon/prism.

## Introduction

Skin cancer is the most frequently diagnosed group of cancers worldwide, with 1.67 million cases estimated in 2025^1^. The annual incidence of skin cancer in the United States exceeds the combined incidence of all other malignancies^2^. Moreover, accurate diagnosis depends on the expertise of specialized professionals, who are often not readily available in emerging countries and remote areas. In Brazil, for example, only 0.2% of the dermatologists practice in cities with fewer than 100,000 inhabitants, whereas 60.3% are concentrated in the capital^3^. Due to its high incidence and shortage of qualified professionals, skin cancer poses a substantial burden to healthcare systems worldwide.

Over the past decades, many Computed Aided Diagnostic (CAD) systems have been proposed to promote access to skin cancer diagnostic^4^. Pioneer works were primarily based on manual feature engineering and traditional Machine Learning (ML) methods. Celebi et al.^5^ extracted texture and color features from dermoscopy images, using the Gray Level Co-occurence Matrix (GLCM) and statistics over color spaces. Those features were further used as input to a Support Vector Machine (SVM) classifier. Ballerini et al.^6^ proposed a K-Nearest Neighbor-based skin cancer classification method using colour and texture features. Correa et al.^7^ used various image processing techniques, such as dilation, erosion, Otsu algorithm^8^, and CLAHE^9^ to extract ABDC rule-based features^10^ from dermoscopic images. Later, an SVM classified the lesions into malignant or benign.

In the last decade, with the increase in computational power, Convolutional Neural Networks (CNNs) have proven effective in either replacing or augmenting handcrafted features and improved classification performance. Esteva et al.^11^ and Han et al.^12^ achieved results comparable to dermatologists using the GoogleNet Inception V3^13^ and ResNet-50^14^ architectures, respectively. CAD systems have also benefited from the development of attention mechanisms. Nazazi et al.^15^ integrated Spatial Group-wise Enhance^16^ and Convolutional Block Attention Module^17^ into an EfficientNet-B3^18^ and achieved results comparable to large CNN ensemble models. Alongside these classification improvements, advanced deep learning paradigms–such as semi-supervised frameworks–continue to drive state-of-the-art progress across broader medical image analysis tasks^19,20^.

More recently, methods that integrate patient metadata have gained popularity, particularly due to the availability of datasets such as PAD-UFES-20^21^, Derm7pt^22^, and ISIC-2019^23^^24^^25^. Pacheco et al.^26^ used clinical data to generate attention maps and select the best features extracted from CNNs. Their feature fusion method, named MetaBlock, improved the results over basic feature concatenation and vanilla CNN baselines. Ou et al.^27^ introduced a cross-attention-based fusion module to allow metadata and image features to influence each other. He et al.^28^ modeled CNN predictions and metadata as nodes in a Bayesian Network to improve skin lesions diagnosis. Xu et al.^29^ proposed the RemixFormer++ a multimodal classifier that also employs a cross-attention-based feature fusion module. Their approach outperformed 191 physicians, most of whom were board-certified dermatologists. Bouzon et al. introduced the MetaBlock-SE, an adaptation of the MetaBlock architecture based on sentence embeddings. Their method demonstrated superior performance when evaluated on datasets with missing patient information. Jin et al.^30^ proposed an ensemble of Naive Bayes and ResNet-18^14^ to classify malignancy in skin diseases. The Naive Bayes was trained on metadata, whereas the CNN on images. Latter, the individual predictions were jointly aggregated by different weights.

Despite recent advances, many feature fusion methods lack mechanisms to transparently adapt to varying amounts of metadata available at inference time. This limitation hinders the interpretability of diagnostic models: clinicians are unable to observe how predictions evolve as patient information is incrementally introduced, making it difficult to trace the influence of specific features on lesion assessment. Enhanced interpretability would allow medical practitioners to interpret model behavior, validate clinical relevance, and selectively focus on meaningful metadata subsets. To address this, we propose PRISM: Probabilistic Reasoning Interpretable Stepwise Model, an interpretable fusion framework that integrates vision model predictions with patient metadata using the simplest form of Bayesian Network–Naive Bayes^31^. Unlike standard ensemble methods (e.g., Jin et al.^30^) that compute image and metadata predictions independently and average them, PRISM incorporates the image prediction directly as a Bayesian prior. This distinction ensures that metadata acts to refine the visual assessment rather than compete with it. This design preserves baseline vision performance when metadata are absent, while also providing a clear and interpretable probabilistic structure that reveals how each metadata element contributes to the final diagnosis. The main contributions of this paper are:

- Introduction of PRISM, a multimodal skin cancer classification framework that provides stepwise interpretability. This allows clinicians to observe and quantify the impact of each clinical feature on the model’s evolving diagnostic prediction, thereby fostering trust and enabling detailed clinical reasoning.
- Implementation of a Stepwise Calibration protocol to dynamically correct the compounding overconfidence inherent in conditional independence assumptions. By tailoring the temperature scaling penalty to the exact volume of integrated clinical metadata, this approach maintains statistically reliable intermediate probability distributions and prevents the severe miscalibrations caused by standard global scaling.
- A feature fusion methodology demonstrating competitive or superior diagnostic performance compared to larger, state-of-the-art multimodal approaches.
- A computationally lightweight method designed for efficient deployment, where resource constraints are primarily dictated by the complexity of the integrated vision backbone rather than the fusion mechanism itself.
- Extensive validation across three diverse skin cancer datasets, including a proprietary, biopsy-proven cohort comprising over sixteen thousand clinical images.
- An open-source implementation of the PRISM framework, available at: https://github.com/life-ufes/prism/.

## Methods

This section details the experimental methodology and our proposed method, PRISM: Probabilistic Reasoning Interpretable Stepwise Model. PRISM is a multimodal fusion framework designed to provide stepwise interpretability, mirroring clinical diagnostic reasoning. It leverages the probabilistic structure of a Naive Bayes classifier to transparently integrate deep vision model predictions with patient metadata, addressing the black-box limitations of common fusion techniques.

### Background: Bayesian classification

A Bayesian Network is a graphical model that describes a set of conditional independence assumptions about a probability distribution. It is represented by a directed acyclic graph (DAG) where each node is a random variable and edges represent the influence of nodes on each other. Such a graph encodes the belief that each node is conditionally independent of its non-descendants given its parents^31^.

Let $\textbf{Y}$ and $\textbf{X}$ be random variables that describe the diagnosis and features, respectively. In classification, the goal is to find the most probable diagnosis $y_j$ given the observed features $\textbf{X}$, which is represented by the posterior distribution $P(\textbf{Y} | \textbf{X})$. This distribution is calculated using Bayes’ Theorem, which combines the likelihood $P(\textbf{X} | \textbf{Y})$, the prior $P(\textbf{Y})$, and the evidence $P(\textbf{X})$ as seen in equation (1).

1 $$\begin{aligned} P(\textbf{Y} | \textbf{X}) = \frac{P(\textbf{X} | \textbf{Y}) \cdot P(\textbf{Y})}{P(\textbf{X})} \end{aligned}$$

For classification, we use a maximum a posteriori (MAP) decision rule to select the most likely diagnosis $\hat{y}$ (equation (2)). Since $P(\textbf{X})$ is a constant normalizing factor for all classes, it can be omitted from the maximization, simplifying the decision.

2 $$\begin{aligned} \hat{y}&= \underset{j}{\arg \max }\ \ P(y_j | \textbf{X}) \propto \underset{j}{\arg \max }\ \ P(\textbf{X} | y_j) \cdot P(y_j) \end{aligned}$$

The primary challenge in Bayesian classification is estimating the likelihood $P(\textbf{X} | \textbf{Y})$. In a general Bayesian Network, this involves defining and learning the conditional probability distribution for each node given its parents. For a complex, densely connected graph, estimating these probabilities can be computationally intractable, demanding an exponential amount of data to accurately learn all joint distributions, especially with many parent nodes^31^.

The Naive Bayes model simplifies this by assuming that all individual features $\textbf{X} = [x_1,..., x_N]$ are conditionally independent given the diagnosis $\textbf{Y}$. In the context of clinical data, this strong independence assumption is frequently violated. Patient metadata and clinical features are intrinsically correlated; for instance, advancing age naturally correlates with specific comorbidities, and various physiological symptoms frequently co-occur for a given pathology. Despite these dependencies, Naive Bayes remains empirically effective for classification tasks. As demonstrated by Domingos and Pazzani^32^, classification accuracy relies on the position of the decision boundary rather than the precise calibration of the estimated probabilities. While correlated features may artificially inflate the combined likelihoods–often pushing the unnormalized posterior estimates toward extreme values of 0 or 1–the relative rank order of the target classes is typically preserved. Consequently, as long as the most probable class maintains the highest relative score, the model will output the correct prediction despite the violated assumptions.

This assumption allows the likelihood to factorize for a given diagnosis $y_j$ as shown in equation (3).

3 $$\begin{aligned} P(\textbf{X} | y_j) = \prod _{i=1}^{N}P(x_i | y_j) \end{aligned}$$

Replacing this factorization into the MAP rule gives the final Naive Bayes classifier, as seen in equation (4).

4 $$\begin{aligned} \hat{y} = \underset{j}{\arg \max }\ \ P(y_j) \prod _{i=1}^{N}P(x_i | y_j) \end{aligned}$$

The estimation of the likelihood distribution $P(\textbf{X} | \textbf{Y})$ follows distinct approaches based on the type of data. For categorical features, standard Multinomial Naive Bayes estimates probabilities based purely on class-conditional frequencies. However, real-world clinical datasets are inherently imbalanced. Relying on standard frequency counts would bias the likelihood estimations toward prevalent classes.

To address this, we introduce a modified, balanced likelihood formulation. Instead of a standard generative likelihood, we estimate $P(x_{i} = k | y_j)$ as the prevalence of the *k*-th value for the *i*-th feature in class $y_j$ relative to its weighted prevalence across all classes. Let $count(x_{i} = k, y_j)$ be the number of occurrences where the *i*-th variable takes the specific value *k* for diagnosis $y_j$. The class-imbalanced adjusted likelihood is formulated in equation (5):

5 $$\begin{aligned} P(x_{i} = k | y_j) = \frac{ count(x_{i} = k, y_j) \cdot W_j }{ \sum _{c=1}^{C} \left( count(x_{i} = k, y_c) \cdot W_c \right) + \epsilon } \end{aligned}$$

where $W_j = \frac{\sum _{l=1}^{C}count(y_l)}{count(y_j)}$ acts as an inverse frequency weight for class $y_j$. This formulation provides an intuitive balance: it scales up the impact of observations from minority classes and penalizes those from majority classes, ensuring that the feature likelihood reflects true class association rather than mere sample prevalence.

The parameter $\epsilon$ is a small additive constant functioning as Laplace smoothing. It ensures that when a certain feature value is entirely absent from the training data for all classes, the denominator remains non-zero, preventing undefined operations and numerical instability. Furthermore, if a categorical feature value is missing for a given patient during inference, the likelihood $P(x_{i} = k | y_j)$ is uniformly set to 1 for all classes $y_j$. This effectively omits the missing feature from the product in equation (4), ensuring that only observed clinical evidence updates the probabilistic prediction.

For numerical features, such as patient age, the probability distributions are modeled as Normal distributions, which is the core assumption of the Gaussian Naive Bayes classifier. Continuous variables were utilized in their original scale without prior normalization, preserving their direct clinical interpretability. The mean and variance are directly estimated from the raw training data for each feature and diagnosis combination. Equation (6) shows the likelihood estimation where $x_i$ is the i-th numerical feature, $y_j$ is the j-th diagnosis, and $\mu _{_{i,j}}$ and $\sigma _{_{i,j}}^2$ are the estimated mean and variance of $x_i$ given $y_j$.

6 $$\begin{aligned} P(x_{i} | y_j) = \frac{1}{\sqrt{2\pi \sigma _{_{i,j}}^2}} \exp \left( -\frac{(x_i - \mu _{_{i,j}})^2}{2\sigma _{_{i,j}}^2} \right) \end{aligned}$$

Consistent with the handling of categorical data, if a numerical feature is absent for a given patient during inference, its likelihood $P(x_{i} | y_j)$ is uniformly set to 1 for all classes $y_j$. This effectively omits the missing numerical feature from the stepwise probability update.

### PRISM: Probabilistic Reasoning Interpretable Stepwise Model

The key mechanism of PRISM is the construction of an audit trail of the model’s reasoning using the Naive Bayes classifier for iterative probability refinement. This is achieved by first training a deep vision model on lesion images. The resulting softmax probability distribution is then used not as a final answer, but as an evidence-based prior probability $P(\textbf{Y})$ for the Bayesian framework. This initial estimate reflects the dermatologist’s preliminary assessment based solely on the visual examination of the lesion.

Formally, let $P_0(y_j)$ denote the initial vision-based prior for class $y_j$, which is directly initialized using the softmax probability output $\hat{Y}_{img}^{(j)}$ from the deep vision model:

7 $$\begin{aligned} P_0(y_j) = \hat{Y}_{img}^{(j)} \end{aligned}$$

Suppose we introduce a sequence of *T* available patient metadata observations, denoted as $\{e_1, e_2, \dots , e_T\}$. Each $e_t$ represents the specific clinical evidence introduced at step *t*, corresponding to either an observed categorical state (e.g., $x_i = k$) or a continuous numerical measurement (e.g., $x_i$). At each step $t \in \{1, \dots , T\}$, the probability distribution is iteratively updated using the likelihood of the newly observed evidence $e_t$. The updated posterior probability $P_t(y_j)$ for diagnosis $y_j$ at step *t* is computed as:

8 $$\begin{aligned} P_t(y_j) = \frac{ P(e_t | y_j) \cdot P_{t-1}(y_j) }{ \sum _{c=1}^{C} \left( P(e_t | y_c) \cdot P_{t-1}(y_c) \right) } \end{aligned}$$

where $P(e_t | y_j)$ is the likelihood of the specific evidence $e_t$, calculated using equation (5) for categorical features or equation (6) for numerical features. The denominator is the explicit normalization factor over all *C* classes. This normalization guarantees that $\sum _{j=1}^{C} P_t(y_j) = 1$ at every step. Consequently, the output at any step *t* is a strictly valid probability distribution, rather than an unnormalized score. Provided the model has been properly calibrated, this allows clinicians to interpret the intermediate values as true diagnostic confidence percentages that evolve seamlessly as new evidence is sequentially integrated. This explicit recursive formulation creates the verifiable stepwise diagnostic process, closely resembling how clinicians reason in practice.

In our computational implementation, to prevent numerical underflow caused by the continuous multiplication of small conditional probabilities, the operations in equation (8) are performed in log-space. Probability multiplications are executed as log-probability additions, and the softmax function is subsequently applied to retrieve the normalized posterior distributions.

**Fig. 1:**
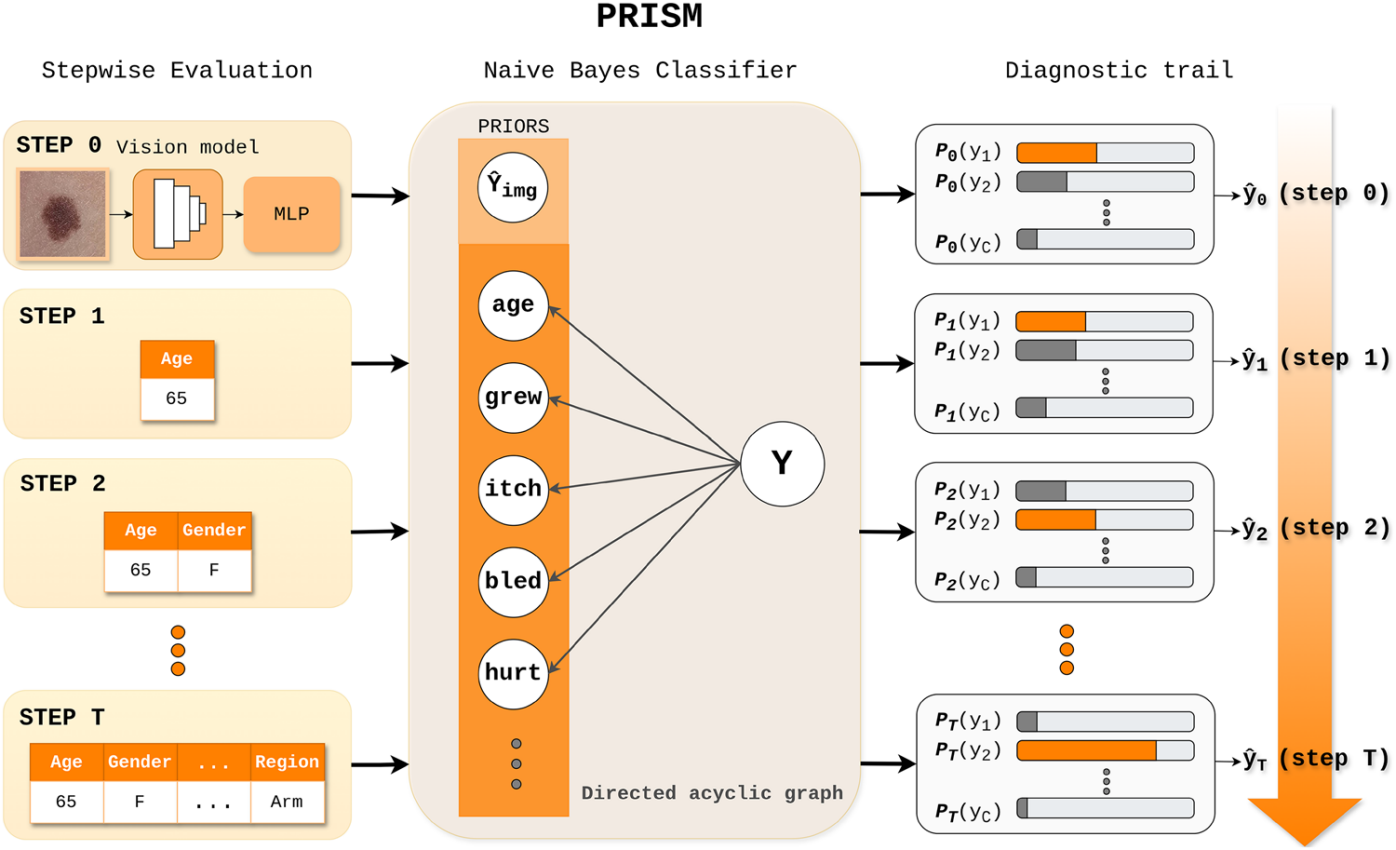
Overview of the PRISM framework. In Step 0, a vision model processes an image to generate an initial visual prediction ($\hat{Y}_{img}$), establishing the initial prior probabilities ($P_0(y_j)$) for each diagnostic class $y_j$, where $j \in \{1, \dots , C\}$. Clinical metadata (e.g., age, gender) is then sequentially introduced (Steps 1 to *T*) into the Naive Bayes Classifier. This sequential integration updates the current diagnostic probability at each step, yielding refined posterior probabilities ($P_t(y_j)$) that form an explicit trail of the model’s evolving reasoning.

Figure 1 provides an overview of the PRISM framework. Initially (Step 0), a lesion image is processed by a deep vision model, and its output is passed through a Multi-Layer Perceptron to yield the preliminary probabilistic prediction $\hat{Y}_{img}$. This establishes the vision-based prior probabilities within the Bayesian classifier, denoted as $P_0(y_j)$ for each diagnostic class $y_j$ where $j \in \{1, \dots , C\}$, representing the initial assessment based solely on visual evidence. Subsequently, clinical metadata is introduced in a stepwise manner. At each step *t*, a subset of patient metadata is incorporated into the Naive Bayes model. Governed by the conditional independence assumption represented in the directed acyclic graph, this sequential integration dynamically updates the diagnostic prediction, resulting in refined posterior probabilities $P_t(y_j)$. The sequence of these progressively updated distributions, from the initial vision prior $P_0(y_j)$ through $P_1(y_j), \dots , P_T(y_j)$, forms an explicit diagnostic trail, providing a transparent record of the model’s evolving reasoning.

A number of clinically and technically advantageous characteristics arise from such a model. First, PRISM’s design provides a direct mechanism for stepwise interpretability, which is a notable distinction from other fusion methods. Because the posterior probability is calculated as a product of likelihoods (equation (3)), clinicians can observe how the diagnostic probability evolves as each piece of metadata is incrementally introduced. For instance, a user can begin with the vision-only prior $P_0(\textbf{Y})$ from the image. By adding the age feature as the first piece of evidence $e_1$ (step $t=1$), they can observe the updated distribution $P_1(\textbf{Y})$. Subsequently, adding the lesion region as evidence $e_2$ (step $t=2$) yields the further refined posterior $P_2(\textbf{Y})$. This stepwise evaluation reveals the impact of each clinical feature, directly aligning with a physician’s diagnostic reasoning. This level of granular, step-by-step interpretability is not achievable with end-to-end models like MetaBlock^26^, which require a complete input vector and whose fusion mechanisms are opaque.

Second, this design addresses a challenge in clinical practice that is frequently overlooked in current literature: the handling of incomplete patient records. While missing data is a standard occurrence in real-world clinical workflows, few multimodal methods explicitly account for it without relying on artificial imputation. A direct consequence of this formulation is that the framework is inherently robust to missing data. In equation (3), we only multiply the likelihoods for the metadata features that are provided. For example, if a patient’s lesion region is unknown, that term is simply omitted from the product in equation (3) for that patient. There is no need for complex encoding or data imputation. This is an advantage over attention-based methods, such as Cross-Attention^27^ and Cross Modality Fusion^33^, which rely on fixed-size input vectors and require encoding strategies that can introduce bias or mask the true influence of missing information. Therefore, if no metadata is provided, the final prediction reduces to the vision-only prior $P_0(\textbf{Y})$, meaning the results are the same as the vision model alone.

Third, the number of parameters added to the vision model is exceptionally small. Each numerical feature incurs 2*C* parameters, and a categorical variable with *k* possible values adds *kC* parameters, where *C* is the number of distinct diseases.

Finally, the framework is agnostic to the vision model: the backbone can be replaced without impacting the Bayesian classifier learned parameters. For instance, a health system could upgrade its vision backbone from a lightweight MobileNet^34^ to a large ensemble model. The new model’s softmax outputs can be fed directly into the existing PRISM framework, as the learned metadata likelihood distributions remain valid and do not need to be retrained. Furthermore, this modularity means the image-based prior could be generated from a different modality entirely (e.g., switching from a model trained on clinical photos to one trained on dermoscopic images) without altering the learned metadata likelihoods.

### Datasets

#### PAD-UFES-20

The PAD-UFES-20 is a publicly available dataset that contains clinical images of six skin lesions: Melanoma (MEL), Actinic Keratosis (ACK), Basal Cell Carcinoma (BCC), Squamous Cell Carcinoma (SCC), Melanocytic Nevi (NEV) and Seborrheic Keratosis (SEK). Supplementary Figure S2 provides representative examples for each diagnostic category. Altogether, there are 2,298 samples from 1,373 patients, which were collected by the Dermatological and Surgical Assistance Program of the Federal University of Espìrito Santo (PAD-UFES, Brazil), a nonprofit initiative that provides free skin lesion treatment to underserved populations, primarily low-income and rural individuals.

Furthermore, each image is accompanied by up to 21 patient metadata attributes with demonstrated relevance for skin cancer classification^35^. These comprise patient demographics (age, gender, skin type via Fitzpatrick scale), behavioral and environmental exposures (smoking, drinking, pesticide exposure), socio-economic indicators (access to piped water, access to a sewer system), family medical history (skin cancer, any cancer, maternal and paternal ancestry), and lesion-specific clinical characteristics (region, elevation, first and second diameters, and whether the lesion grew, itch, bled, hurt, or changed). Through exploratory data analysis, we selected ten these for our model. The single numerical feature, age, was modeled using a Gaussian Naive Bayes approach. The remaining nine categorical and binary features–skin cancer history, any cancer history, lesion region, itch, grew, hurt, changed, bled, and elevation–were handled by a Multinomial Naive Bayes model. In the implementation of the state-of-the-art methods, we used the same features as the original authors.

#### PAD-UFES-20+

The PAD-UFES-20+ is an expanded version of the PAD-UFES-20 that comprises 16,318 samples from 6,063 patients. Data were collected prospectively and directly from patients between 2019 and 2025, in accordance with the Brazilian National Health Council (CNS) Resolution No. 466/2012. As mandated by these guidelines, written informed consent was obtained from all subjects and/or their legal guardian(s) prior to data acquisition. Supplementary Table S1 presents a comparison of the number of samples per class in both datasets, while Supplementary Figure S3 exemplifies the diagnostic categories through representative clinical images. The PAD-UFES-20+ contains the same set of clinical information as the PAD-UFES-20, except for the diameters, which were not further collected. However, a key distinction lies in the data heterogeneity and the amount of missing information, particularly for the features grew, hurt, bled, elevation, changed, and itch. Supplementary Figure S1 illustrates the percentage of missing or unknown values for each feature, highlighting a marked disparity in data completeness between the two datasets. Consequently, the PAD-UFES-20+ presents a distinct challenge for multimodal skin cancer classifiers compared to the original benchmark.

#### MILK10k

The MILK10k is a multi-centric skin cancer dataset collected during routine dermatological care across institutions in Austria, Turkey, the United States, North Macedonia, and Australia. It comprises 10,480 images organized into 5,240 pairs, where each lesion is represented by a clinical close-up and its matching dermoscopic counterpart. The dataset includes samples from the following diagnostic categories: Basal cell carcinoma (BCC), Melanocytic nevus (NV), Benign keratinocytic lesion (BKL), Squamous cell carcinoma/keratoacanthoma (SCCKA), Melanoma (MEL), Actinic keratosis/intraepidermal carcinoma (AKIEC), Dermatofibroma (DF), Inflammatory and infectious lesions (INF), Vascular lesions and hemorrhage (VASC), Other benign proliferations including collisions (BEN_OTH), and Other malignant proliferations including collisions (MAL_OTH). Supplementary Figure S4 illustrates the morphological diversity of the dataset, providing representative examples for each diagnostic category.

Supplementary Table S2 shows the number of samples per diagnostic. Although the dataset presents a challenging imbalance–with some classes containing fewer than 10 samples–we opted to retain the original diagnostic granularity rather than aggregating minority classes. This ensures our evaluation remains consistent with the standard protocols established by the dataset creators^36^, preserving the distinction of rare pathologies. In addition to image data, MILK10k provides patient-level metadata, including approximate age, anatomical site, skin tone, and gender. Each image is also annotated with concept term probabilities derived from the MONET framework^37^, covering the following concept groups: (i) ulceration and crust, (ii) hair, (iii) vasculature and vessels, (iv) erythema, (v) pigmentation, (vi) gel, water drop, fluid, and dermoscopy liquid, and (vii) skin markings, pen ink, and purple pen.

### Experimental setup

To generate the visual priors for our Naive Bayes classifier, we employed four modern deep learning backbones, selected to represent two distinct architectural paradigms. Specifically, we utilized EfficientNet-B0^18^ and MobileNet-V3^34^ to represent Convolutional Neural Networks (CNNs), alongside DaViT-Tiny^38^ and SwinV2-Tiny^39^ to represent Vision Transformers. These foundational architectures inherently serve as our single-modality (vision-only) baselines. Furthermore, to rigorously evaluate PRISM’s integration mechanism, we benchmarked it against four state-of-the-art multimodal feature-fusion methods: MetaBlock-SE^40^, a multimodal Bayesian Network^28^, Cross-Attention^27^, and Cross Modality Fusion^33^.

The deep learning models were implemented using PyTorch, pre-trained on ImageNet 1 K with weights sourced from the TIMM API, and fine-tuned on the target datasets for 100 epochs on an NVIDIA RTX A6000 GPU. Training was conducted using the Adam^41^ optimizer with exponential decay rates $\beta _1 = 0.9$ and $\beta _2 = 0.999$, paired with the 1cycle learning rate policy^42^. To ensure a strictly controlled comparison, a standardized optimization protocol was applied to the vision-only baselines and all deep learning architectures operating on tabular metadata. Under this unified protocol, the learning rate was cycled from an initial value of $10^{-7}$ up to a maximum rate of $10^{-5}$ during a warm-up phase lasting 20% of the total steps, before being cosine-annealed back down to $10^{-7}$. In accordance with the 1cycle policy, momentum was also cycled, varying inversely with the learning rate. Early stopping was applied when the validation loss exhibited no improvement for 10 consecutive epochs.

Exceptions were strictly made to this unified protocol to accommodate the unique dynamics of specific baselines and faithfully reproduce the original authors’ methodologies. Because the MetaBlock-SE baseline relies on a fundamentally distinct NLP-based architecture, we adopted its recommended schedule, cycling from an initial $10^{-6}$ up to a maximum of $10^{-4}$. Similarly, the multimodal Bayesian Network^28^ was implemented in Pyro and trained according to its original protocol: parameter learning was performed via Maximum Likelihood Estimation by optimizing the Evidence Lower Bound (ELBO) loss with the Adam optimizer at a learning rate of $2.5 \times 10^{-3}$, and model selection utilized early-stopping regularization based on validation balanced accuracy. To address class imbalance, we employed a weighted cross-entropy loss function, assigning weights inversely proportional to label frequencies. All input images were resized to 224x224 pixels, and standard data augmentation techniques were applied, including horizontal and vertical flips, adjustments to brightness, contrast, and saturation, image scaling, and the addition of random noise^35,43^.

The Naive Bayes classifier was also implemented in PyTorch. The conditional probabilities for categorical and numerical features were estimated using equations (5) and (6), respectively. Importantly, the “No Metadata” vision-only baselines evaluated in our experiments represent the exact, unaltered probabilistic priors fed into the framework. This design inherently functions as an ablation study, explicitly isolating the performance contribution of the clinical metadata integration from the baseline vision predictions. Finally, class assignments were determined using Maximum a Posteriori queries, as described in equation (4).

All experiments were conducted using 5-fold cross-validation, stratified by label frequency while grouping by patient to ensure unbiased estimations. To prevent data leakage, the vision backbone and the subsequent Naive Bayes parameters were trained sequentially and strictly isolated within each fold’s training partition. Model effectiveness was assessed using the mean and standard deviation of balanced accuracy (BACC), F1-score, area under the curve (AUC), Specificity, and Precision. Given the substantial class imbalance present in the datasets, BACC – defined as the macro-average of recall (sensitivity) obtained on each class – was adopted as the primary evaluation metric.

To statistically compare the experimental results and explicitly address the non-independence of repeated cross-validation folds across diverse architectures, for each dataset, the 120 total runs (6 evaluated approaches $\times$ 4 backbones $\times$ 5 folds) were evaluated using a Linear Mixed-Effects Model (LMM). As demonstrated by Corani et al.^44^, hierarchical modeling–such as the mixed-effects approach employed here–is a statistically rigorous framework for algorithm benchmarking, as it correctly accounts for the nested correlation structures inherent in cross-validation. Prior to modeling, the evaluation metric was logit-transformed to satisfy parametric assumptions. Specifically, the LMM was formulated as:

$$\begin{aligned} Y_{ijk} = \beta _0 + \beta _i + u_j + v_k + \epsilon _{ijk} \end{aligned}$$

where $Y_{ijk}$ represents the logit-transformed performance metric for the *i*-th evaluated approach applied to the *j*-th vision backbone during the *k*-th cross-validation fold. In this formulation, $\beta _0$ is the global intercept, and $\beta _i$ is the fixed main effect of the *i*-th approach. To account for repeated measures, the vision backbones and cross-validation folds were modeled as crossed random effects, represented by the random intercepts $u_j \sim \mathcal {N}(0, \sigma _u^2)$ and $v_k \sim \mathcal {N}(0, \sigma _v^2)$, respectively. The term $\epsilon _{ijk} \sim \mathcal {N}(0, \sigma ^2)$ denotes the residual error. Because our objective was to evaluate the generalized performance contribution of the fusion methodology across varying contexts, no interaction terms (e.g., method $\times$ backbone) were included in the formulation.

Following a significant Wald omnibus test, post-hoc pairwise comparisons were conducted via contrasts of the Estimated Marginal Means (EMMs), with our proposed method designated as the control against each baseline. To strictly control the Family-Wise Error Rate across multiple comparisons, all resulting *p*-values were adjusted using the Holm step-down procedure^45^, with significance established at $\alpha = 0.05$.

### Ethical approval

This study was approved by the Ethics Committee of the Federal University of Espìrito Santo and by the Brazilian government through the Plataforma Brasil (CAEE No. 78728324.3.0000.5060). All data were collected with the patients’ consent.

## Results

This section presents a comprehensive evaluation of the PRISM framework. We begin by presenting the diagnostic performance of our proposed method, demonstrating its competitive or superior capabilities against existing state-of-the-art multimodal approaches on three skin cancer benchmarks. Following this, we provide a detailed investigation of PRISM’s interpretability. Through four illustrative case studies, we experimentally demonstrate how PRISM facilitates step-wise reasoning, offering enhanced transparency into the model’s decision-making process, a distinct advantage from conventional black-box methods.

### Benchmark performance analysis on PAD-UFES-20

In this experiment, we analyze the performance of the proposed model on the PAD-UFES-20 dataset. To ensure a fair interpretation of the results, Supplementary Table S3 details the specific metadata inputs available to each method. For the baseline architectures–MetaBlock-SE^40^, Bayesian Network^28^, and Cross-Attention^27^–we strictly employed the identical feature sets defined in their original publications. Because the authors of the Cross Modality Fusion^33^ method did not explicitly specify their utilized clinical variables, we evaluated it using the same comprehensive feature set as the other attention-based models to ensure a rigorous and equivalent comparison. Moreover, it should be noted that, unlike He et al.^28^, we did not impute missing metadata during preprocessing. Instead, we relied on the Bayesian Network’s intrinsic capability to handle missing information directly within the inference process. Conversely, the feature set for the proposed PRISM framework was selected after exploratory data analysis.

**Fig. 2:**
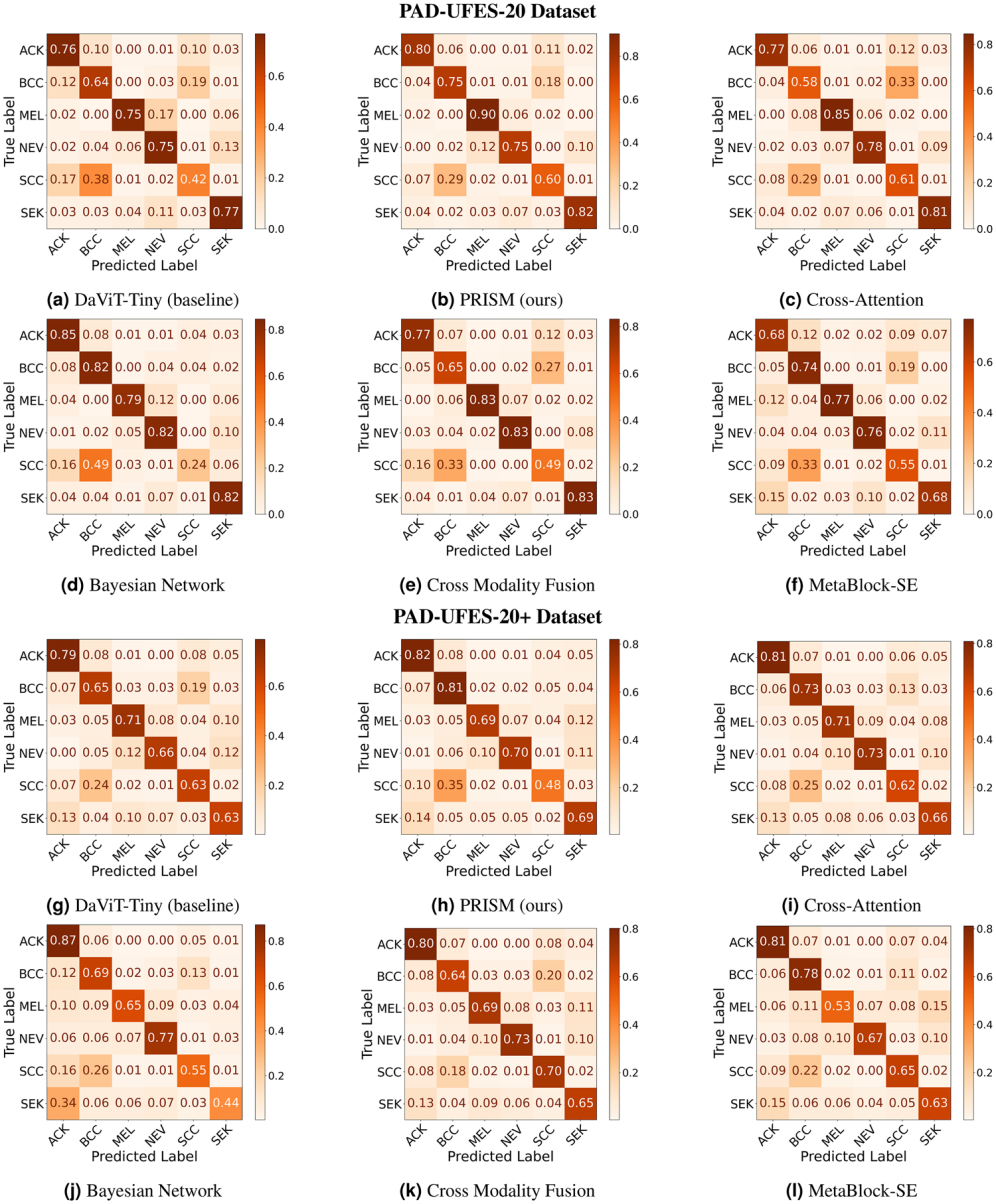
Comparison of cross-validation confusion matrices across datasets. **(A) PAD-UFES-20 dataset:** The baseline DaViT-Tiny model **(a)** is compared against the proposed PRISM framework **(b)**, as well as state-of-the-art fusion techniques: **(c)** Cross-Attention^27^, **(d)** Bayesian Network^28^, **(e)** Cross Modality Fusion^33^, and **(f)** MetaBlock-SE^40^. **(B) PAD-UFES-20+ dataset:** The identical model configuration is shown in **(g)** through **(l)**. The diagonal elements represent the recall (sensitivity) for each skin lesion class, where darker colors indicate higher values.

Table 1 presents the cross-validation results under these configurations. The Wald omnibus test indicated highly significant differences among the evaluated methods across all classification metrics (all $p < 0.001$). Post-hoc pairwise comparisons revealed that PRISM outperformed the MetaBlock-SE ($p < 0.001$), Cross Modality Fusion ($p < 0.001$), Bayesian Network ($p < 0.001$), Cross-Attention ($p < 0.001$), and baseline vision models ($p < 0.001$). Furthermore, our method achieved up to 0.772 ± 0.036 balanced accuracy, 0.714 ± 0.015 F1-score, and 0.948 ± 0.011 AUC with the DaViT-Tiny backbone. Additionally, Fig. 2a–f display the confusion matrices for the DaViT-Tiny backbone standalone and combined with the fusion methods. Except for NEV, where baseline sensitivity was maintained, our approach improves performance across all classes. Most notably, PRISM increases MEL sensitivity from 0.75 to 0.90 compared to the vision baseline.

**Table 1:** Classification performance on the PAD-UFES-20 dataset. The proposed PRISM framework is compared against vision-only baselines (No Metadata) and state-of-the-art multimodal fusion methods across four vision backbones. Values are reported as mean ± standard deviation.

Model	BACC	F1-Score	AUC	Specificity	Precision
No Metadata					
DaViT-Tiny	$0.677\pm 0.019$	$0.650\pm 0.009$	$0.924\pm 0.014$	$0.933\pm 0.005$	$0.640\pm 0.009$
EfficientNet-B0	$0.615\pm 0.029$	$0.591\pm 0.027$	$0.896\pm 0.019$	$0.923\pm 0.004$	$0.589\pm 0.032$
MobileNet-V3	$0.622\pm 0.059$	$0.592\pm 0.048$	$0.891\pm 0.023$	$0.921\pm 0.011$	$0.587\pm 0.045$
SwinV2-Tiny	$0.639\pm 0.029$	$0.619\pm 0.026$	$0.906\pm 0.014$	$0.922\pm 0.009$	$0.633\pm 0.034$
PRISM (ours)					
DaViT-Tiny	$0.772\pm 0.036$	$0.714\pm 0.015$	$0.948\pm 0.011$	$0.952\pm 0.005$	$0.704\pm 0.010$
EfficientNet-B0	$0.741\pm 0.048$	$0.668\pm 0.030$	$0.939\pm 0.014$	$0.947\pm 0.005$	$0.665\pm 0.023$
MobileNet-V3	$0.749\pm 0.054$	$0.691\pm 0.042$	$0.939\pm 0.016$	$0.946\pm 0.006$	$0.683\pm 0.034$
SwinV2-Tiny	$0.753\pm 0.046$	$0.703\pm 0.034$	$0.943\pm 0.011$	$0.946\pm 0.008$	$0.699\pm 0.024$
Cross-Attention					
DaViT-Tiny	$0.733\pm 0.051$	$0.674\pm 0.051$	$0.937\pm 0.010$	$0.938\pm 0.008$	$0.673\pm 0.046$
EfficientNet-B0	$0.696\pm 0.018$	$0.668\pm 0.022$	$0.933\pm 0.007$	$0.938\pm 0.005$	$0.670\pm 0.032$
MobileNet-V3	$0.722\pm 0.047$	$0.671\pm 0.035$	$0.930\pm 0.014$	$0.934\pm 0.013$	$0.675\pm 0.024$
SwinV2-Tiny	$0.740\pm 0.044$	$0.692\pm 0.044$	$0.939\pm 0.014$	$0.941\pm 0.010$	$0.684\pm 0.045$
Cross Modality Fusion					
DaViT-Tiny	$0.731\pm 0.032$	$0.704\pm 0.031$	$0.939\pm 0.008$	$0.942\pm 0.006$	$0.705\pm 0.028$
EfficientNet-B0	$0.711\pm 0.028$	$0.672\pm 0.021$	$0.934\pm 0.011$	$0.941\pm 0.005$	$0.661\pm 0.022$
MobileNet-V3	$0.710\pm 0.044$	$0.680\pm 0.028$	$0.931\pm 0.014$	$0.937\pm 0.007$	$0.674\pm 0.029$
SwinV2-Tiny	$0.717\pm 0.034$	$0.674\pm 0.022$	$0.927\pm 0.010$	$0.937\pm 0.007$	$0.664\pm 0.027$
Bayesian Network					
DaViT-Tiny	$0.730\pm 0.055$	$0.697\pm 0.039$	$0.937\pm 0.012$	$0.952\pm 0.007$	$0.691\pm 0.030$
EfficientNet-B0	$0.684\pm 0.025$	$0.651\pm 0.022$	$0.925\pm 0.013$	$0.945\pm 0.006$	$0.651\pm 0.015$
MobileNet-V3	$0.690\pm 0.039$	$0.667\pm 0.040$	$0.927\pm 0.018$	$0.946\pm 0.007$	$0.678\pm 0.036$
SwinV2-Tiny	$0.703\pm 0.040$	$0.677\pm 0.030$	$0.931\pm 0.012$	$0.947\pm 0.008$	$0.677\pm 0.021$
MetaBlock-SE					
DaViT-Tiny	$0.694\pm 0.021$	$0.660\pm 0.011$	$0.915\pm 0.011$	$0.937\pm 0.004$	$0.656\pm 0.024$
EfficientNet-B0	$0.677\pm 0.057$	$0.671\pm 0.053$	$0.917\pm 0.018$	$0.939\pm 0.009$	$0.679\pm 0.045$
MobileNet-V3	$0.668\pm 0.016$	$0.643\pm 0.005$	$0.914\pm 0.013$	$0.936\pm 0.008$	$0.638\pm 0.011$
SwinV2-Tiny	$0.692\pm 0.034$	$0.666\pm 0.046$	$0.916\pm 0.010$	$0.938\pm 0.009$	$0.672\pm 0.040$

While hierarchical Bayesian approaches, such as the multimodal network proposed by He et al.^28^, capture complex dependencies by conditioning the *i*-th metadata feature $x_i$ on both the diagnosis $\textbf{Y}$ and the vision prediction $\hat{Y}_{img}$, this design requires estimating $O(C^2)$ parameters per feature and relies on iterative Evidence Lower Bound (ELBO) minimization to approximate the posterior. In clinical datasets with limited sample sizes, learning these complex joint distributions forces the model to partition the training data into increasingly smaller subsets, leading to severe data sparsity and high parameter variance. Consequently, the complex model is prone to convergence instability and heavily overfits to training noise. In contrast, PRISM employs a simpler topology ($P(x_i | \textbf{Y})$) that scales linearly (*O*(*C*)) and admits closed-form Maximum Likelihood Estimation–calculating frequency counts for Multinomial nodes (equation (5)) and means and variances for Gaussian nodes (equation (6)) directly from the data. Viewed through the lens of the bias-variance tradeoff, PRISM’s strong assumption of conditional independence introduces theoretical bias; however, it drastically reduces parameter variance because the parameters are estimated using the full marginal support of the data for each class. Our results (Table 1) indicate that this reduction in variance outweighs the independence bias, allowing PRISM to yield superior generalization ($p_{bacc} < 0.001$) compared to a theoretically complex but difficult-to-train architecture.

#### Incremental evaluation under distribution shift

**Fig. 3:**
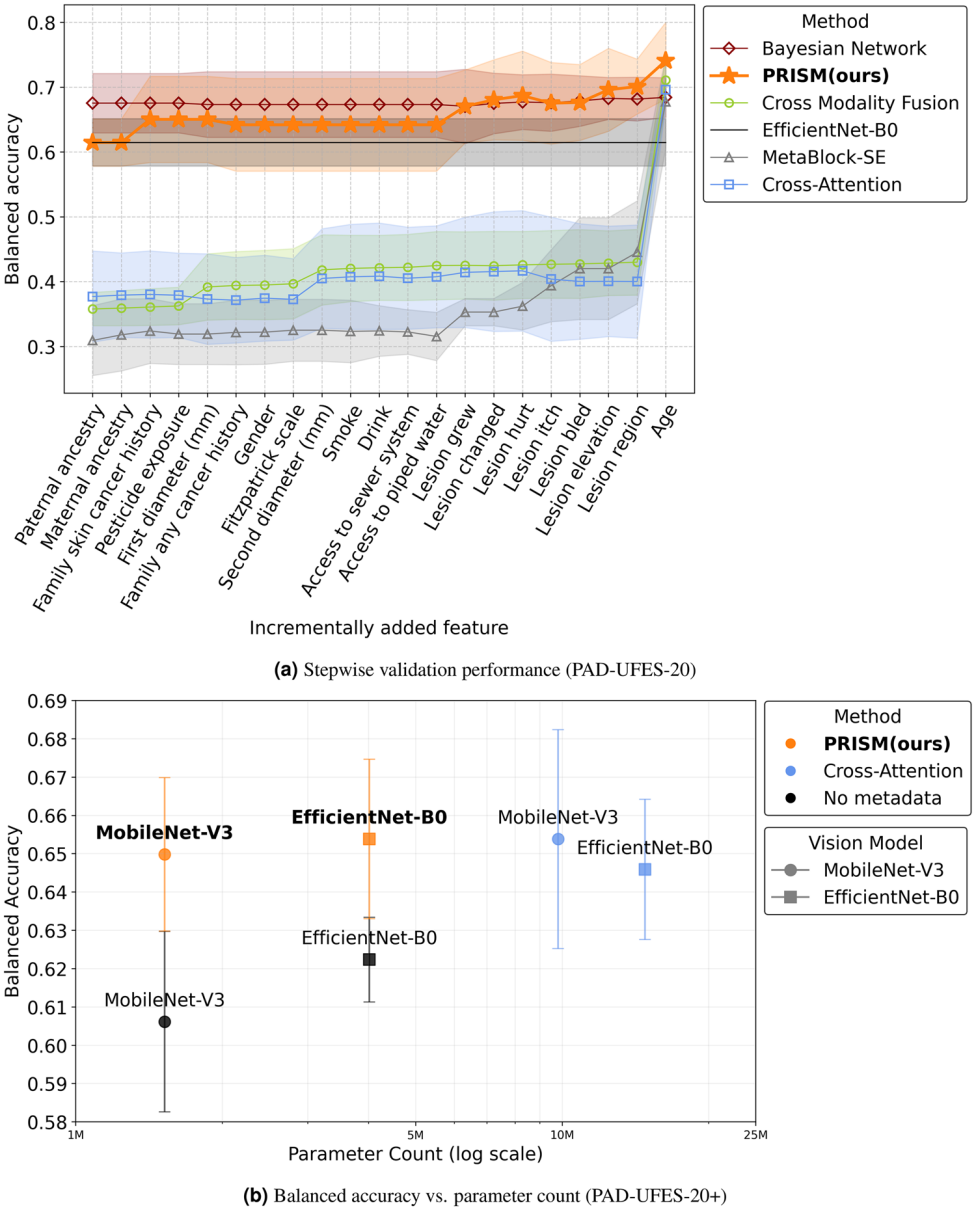
Performance, complexity, and robustness analysis. **(a)** The model is trained on the full set of metadata, while validation introduces features sequentially to simulate a clinical workflow. PRISM ensures accuracy never falls below the vision-only baseline (EfficientNet-B0), unlike alternative attention-based fusion strategies that require complete data. Shaded areas represent the 95% CI computed using the Student’s t-distribution. **(b)** PRISM improves performance over vision-only baselines with negligible parameter overhead, avoiding the substantial complexity increase observed in the Cross-Attention method. Vertical bars represent the 95% CI computed using the Student’s t-distribution.

When integrating clinical data with image-based classifiers, a robust method should ideally maintain, or improve upon, the performance of the vision model even with varying amounts of metadata available. To evaluate this behavior, we designed an experiment where metadata features were incrementally introduced to the validation set, and the classifiers’ performance was assessed at each step. Figure 3a illustrates the balanced accuracy as a function of the progressively added features.

The sequence of feature addition was determined by the global frequency of missing values across the entire dataset, ordered from highest to lowest (i.e., from the rarest feature to the most universally available). To ensure a consistent evaluation framework and allow for direct performance averaging across cross-validation folds, this specific ordering–explicitly listed along the x-axis of Fig. 3a–was kept strictly fixed across all folds. This specific sequence was deliberately chosen to simulate a worst-case clinical scenario. By withholding the most universally available and historically predictive features (such as patient Age and Lesion Region) until the final steps, we systematically deprive the classifiers of the primary contextual anchors they heavily rely upon during training. Because machine learning models naturally learn to weight consistently present variables most heavily, evaluating them initially on rarely observed features forces them to operate under a severe distribution shift and information deficit. This sequence tests a crucial clinical safety requirement: whether a multimodal model can gracefully default to the vision-based prior when primary clinical context is missing, or if the absence of these dominant metadata anchors triggers catastrophic performance degradation.

To ensure a fair and robust comparison against the end-to-end and attention-based baselines (which are typically optimized for complete input vectors), we explicitly trained these models to handle missingness. Specifically, missing data was treated as a distinct, informative state across all input formats. For tabular models, we allocated an explicit “missing” category for all categorical features during the one-hot encoding phase, regardless of whether missing values were present in the training set, and missing numerical features were explicitly encoded with a constant value of −1. Notably, certain highly predictive features, such as patient Age, exhibit 100% completeness in the retrospective training data. Consequently, if such a feature is withheld during the stepwise evaluation, deep learning baselines are mathematically forced to process an out-of-distribution (OOD) imputation value (e.g., −1), pushing the model out of its training manifold. Accordingly, before a tabular feature was added to the incremental evaluation set, we purposely adjusted its categorical “missing” indicator to 1 or its numerical value to −1. Similarly, for the NLP-based MetaBlock-SE baseline, missingness was explicitly integrated into the sentence embeddings. During training, any absent feature was assigned the text value “unknown” within the descriptive input sentence (e.g., “Gender: unknown”). Correspondingly, during the stepwise evaluation, the textual prompt was dynamically updated so that any feature not yet introduced in the sequence was strictly held as “unknown”. Despite this controlled handling, a notable observation is that the probabilistic methods, PRISM and Bayesian Network, are the only ones whose balanced accuracy consistently remains at or above the performance of the vision-only model (EfficientNet-B0, represented by the solid black line), regardless of the severe missingness in the early steps.

This vulnerability is most pronounced in MetaBlock-SE due to its unidirectional attention architecture. MetaBlock-SE relies exclusively on the embedded metadata to generate attention maps that enhance the visual features. Consequently, when the metadata input is dominated by unlearned “unknown” tokens, the resulting attention map degrades into noise. Because this noise is directly multiplied onto the vision backbone’s output, it actively corrupts the only valid clinical signal the model has left. In contrast, models like Cross-Attention utilize bidirectional cross-attention mechanisms. By maintaining a secondary, mirrored pathway–where visual features serve as the query to evaluate the metadata–these architectures distribute the fusion representation, making them slightly less susceptible to the catastrophic visual signal collapse observed in strictly unidirectional gating.

Nevertheless, attention-based fusion methods like Cross-Attention and Cross Modality Fusion exhibit performance that is highly dependent on the completeness of the metadata. Under the primary evaluation sequence, where universally present features like Age are deliberately withheld until the final steps, our global missingness encoding forces the network to process unlearned, out-of-distribution placeholders (e.g., a constant −1 for Age). Because the network never observed these specific missing indicators during training, its performance severely degrades below the vision-only baseline, highlighting an inability to safely process unfamiliar missing inputs.

However, to assess whether this degradation was driven exclusively by these OOD artifacts, we conducted an additional sensitivity analysis using an alternative sequence. Here, we introduced the numerical features first (Age, Diameters), followed by the categorical features ordered from least missing to most missing (Supplementary Figure S5). Under this ordering, the model receives the universally available and highly predictive Age in the very first step–completely avoiding its OOD missing indicator. As expected, PRISM immediately leverages this clinical anchor, remaining stable and consistently above the vision-only baseline from step one. Notably, the attention-based and end-to-end baselines still exhibit severe early-step instability. When provided with only patient Age amidst a vector of “missing” indicators, models like Cross-Attention and Cross Modality Fusion suffer a catastrophic drop in performance, falling well below the vision-only baseline. They only manage to recover and surpass the baseline after all continuous variables are fully integrated.

Importantly, this early-step failure in the alternative sequence cannot be attributed to OOD imputation artifacts. While Age is universally present, the lesion diameters are natively missing in approximately 34% of the training instances; therefore, the explicit missingness encoding for those withheld features was heavily represented during training and is strictly in-distribution. The fact that the deep learning baselines degrade when provided with a valid Age alongside in-distribution missing indicators reveals a deeper vulnerability: a brittle architectural reliance on specific feature co-occurrences. Their learned non-linear dependencies and joint representations struggle to contextualize an isolated piece of clinical evidence without the stabilizing presence of its historically correlated features. Furthermore, while deep learning architectures could theoretically be forced to handle arbitrary sparsity through extensive data augmentation–such as simulating missingness via feature dropout during training–this approach requires anticipating and modeling the exact missingness distributions the model will encounter in deployment. In contrast, PRISM eliminates the need for these complex, missingness-aware training protocols. Because its conditional independence assumption natively supports exact marginalization, missing features are dynamically omitted from the stepwise update, allowing the seamless integration of any amount of clinical evidence without predictions being corrupted by the absence of other variables or OOD imputation values.

### Evaluation under high heterogeneity on the PAD-UFES-20+ extension

In the next phase of our evaluation, we evaluate our method in a more demanding scenario using the PAD-UFES-20+, an expanded version of the PAD-UFES-20. Supplementary Table S4 details the specific clinical inputs provided to each method in this expanded cohort. Following exploratory data analysis, we retained the same feature set employed in the PAD-UFES-20 experiment for PRISM evaluation. Similarly, for the state-of-the-art methods, we retained the same feature set as in the previous experiment except for the descendancies, piped water and sewage information, which were removed due to the amount of missing data, and the diameters, which were not further collected. Table 2 details the performance metrics for each method on the PAD-UFES-20+ dataset. The Wald omnibus test indicated highly significant differences among the evaluated methods across all classification metrics (all $p < 0.001$). Following this, post-hoc pairwise comparisons confirmed that PRISM outperformed the baseline vision models ($p < 0.001$) and the Bayesian Network ($p < 0.001$). This time, however, our method achieved a similar balanced accuracy with the MetaBlock-SE ($p = 0.704$), Cross-Attention ($p = 0.604$) and Cross Modality Fusion ($p = 0.324$).

**Table 2:** Classification performance on the PAD-UFES-20+ dataset. The proposed PRISM framework is compared against vision-only baselines (No Metadata) and state-of-the-art multimodal fusion methods across four vision backbones. Values are reported as mean ± standard deviation.

Model	BACC	F1-Score	AUC	Specificity	Precision
No Metadata					
DaViT-Tiny	$0.678\pm 0.011$	$0.612\pm 0.024$	$0.928\pm 0.006$	$0.941\pm 0.003$	$0.575\pm 0.028$
EfficientNet-B0	$0.622\pm 0.009$	$0.556\pm 0.010$	$0.894\pm 0.003$	$0.929\pm 0.002$	$0.520\pm 0.011$
MobileNet-V3	$0.606\pm 0.019$	$0.543\pm 0.013$	$0.894\pm 0.007$	$0.926\pm 0.004$	$0.509\pm 0.011$
SwinV2-Tiny	$0.661\pm 0.022$	$0.584\pm 0.036$	$0.915\pm 0.010$	$0.936\pm 0.006$	$0.549\pm 0.039$
PRISM (ours)					
DaViT-Tiny	$0.698\pm 0.012$	$0.641\pm 0.020$	$0.937\pm 0.005$	$0.946\pm 0.003$	$0.618\pm 0.021$
EfficientNet-B0	$0.654\pm 0.017$	$0.594\pm 0.014$	$0.918\pm 0.003$	$0.938\pm 0.003$	$0.572\pm 0.014$
MobileNet-V3	$0.650\pm 0.016$	$0.591\pm 0.013$	$0.918\pm 0.004$	$0.936\pm 0.003$	$0.576\pm 0.010$
SwinV2-Tiny	$0.685\pm 0.028$	$0.620\pm 0.037$	$0.930\pm 0.008$	$0.942\pm 0.006$	$0.596\pm 0.035$
Cross-Attention					
DaViT-Tiny	$0.708\pm 0.014$	$0.644\pm 0.014$	$0.935\pm 0.004$	$0.947\pm 0.002$	$0.606\pm 0.017$
EfficientNet-B0	$0.646\pm 0.015$	$0.602\pm 0.027$	$0.920\pm 0.006$	$0.936\pm 0.003$	$0.578\pm 0.042$
MobileNet-V3	$0.654\pm 0.023$	$0.597\pm 0.025$	$0.916\pm 0.005$	$0.936\pm 0.003$	$0.571\pm 0.024$
SwinV2-Tiny	$0.700\pm 0.032$	$0.658\pm 0.013$	$0.936\pm 0.006$	$0.947\pm 0.003$	$0.636\pm 0.011$
Cross Modality Fusion					
DaViT-Tiny	$0.702\pm 0.018$	$0.637\pm 0.017$	$0.934\pm 0.003$	$0.944\pm 0.002$	$0.599\pm 0.017$
EfficientNet-B0	$0.656\pm 0.023$	$0.583\pm 0.018$	$0.913\pm 0.003$	$0.934\pm 0.002$	$0.544\pm 0.016$
MobileNet-V3	$0.670\pm 0.009$	$0.594\pm 0.011$	$0.919\pm 0.003$	$0.936\pm 0.002$	$0.555\pm 0.012$
SwinV2-Tiny	$0.693\pm 0.023$	$0.622\pm 0.019$	$0.930\pm 0.006$	$0.943\pm 0.005$	$0.583\pm 0.017$
Bayesian Network					
DaViT-Tiny	$0.661\pm 0.024$	$0.643\pm 0.026$	$0.930\pm 0.006$	$0.941\pm 0.005$	$0.638\pm 0.026$
EfficientNet-B0	$0.595\pm 0.015$	$0.590\pm 0.011$	$0.906\pm 0.004$	$0.930\pm 0.003$	$0.598\pm 0.020$
MobileNet-V3	$0.588\pm 0.007$	$0.582\pm 0.004$	$0.906\pm 0.006$	$0.927\pm 0.003$	$0.594\pm 0.009$
SwinV2-Tiny	$0.630\pm 0.024$	$0.617\pm 0.023$	$0.923\pm 0.009$	$0.936\pm 0.006$	$0.617\pm 0.022$
MetaBlock-SE					
DaViT-Tiny	$0.678\pm 0.023$	$0.644\pm 0.022$	$0.921\pm 0.005$	$0.948\pm 0.002$	$0.625\pm 0.029$
EfficientNet-B0	$0.657\pm 0.023$	$0.617\pm 0.022$	$0.892\pm 0.010$	$0.939\pm 0.003$	$0.591\pm 0.024$
MobileNet-V3	$0.662\pm 0.024$	$0.600\pm 0.015$	$0.912\pm 0.003$	$0.941\pm 0.003$	$0.565\pm 0.011$
SwinV2-Tiny	$0.680\pm 0.020$	$0.624\pm 0.013$	$0.927\pm 0.004$	$0.946\pm 0.003$	$0.591\pm 0.012$

Furthermore, Fig. 2g–l display the confusion matrices for the DaViT-Tiny vision backbone and the state-of-the-art feature fusion methods. PRISM improves sensitivity for the benign NEV, SEK, and ACK diagnoses. Nevertheless, we observed a decline in SCC performance, primarily due to misclassification as BCC. While the clinical impact of this error is mitigated by the fact that both malignancies typically require excision, it remains a limitation not observed in attention-based methods like Cross-Attention and Cross Modality Fusion. Conversely, MetaBlock-SE shows competitive overall results but displays a concerning drop in MEL accuracy, falling from 0.71 to 0.53. Finally, in this larger dataset characterized by substantial missing metadata, the Bayesian Network^28^ suffers a performance detriment, particularly driven by a decrease in SEK sensitivity.

To further examine the trade-offs between these methods, Fig. 3bpresents the balanced accuracy as a function of the parameter count for both PRISM and Cross-Attention approaches across the lightweight convolutional backbones. For MobileNet-V3, although the performance of both methods is comparable, incorporating the Cross-Attention fusion module adds approximately 8.29 M new parameters to the 1.54 M of the original base model. This substantial overhead may limit its applicability in embedded systems or in ensemble configurations designed for resource-constrained platforms such as smartphones and tablets. For EfficientNet-B0, the Cross-Attention module introduces an additional 10.78 M parameters to the original 4.01 M. In contrast, the PRISM fusion module adds fewer than 200 parameters to the base model, irrespective of the vision backbone employed.

### Generalization capability on the multi-centric MILK10k dataset

In this final evaluation experiment, we evaluate our method on dermoscopic images using the recently introduced MILK10k dataset^36^. To ensure methodological transparency, Supplementary Table S5 details the specific clinical inputs available to each method. For the attention-based and end-to-end baselines (Cross-Attention, Cross-Modality Fusion, and MetaBlock-SE), we provided the maximum available context, incorporating both patient demographics and dermoscopic concept features (e.g., vasculature, erythema). Concept features corresponding to macroscopic clinical images were excluded across all models, as this evaluation strictly utilizes dermoscopic image inputs.

Conversely, for the probabilistic frameworks (PRISM and Bayesian Network), dermoscopic concept features were deliberately omitted. Because these methods mathematically integrate metadata directly with the predictive distribution of the vision backbone, providing visual descriptors alongside the raw image introduces informational redundancy. This “double-counting” of visual evidence violates the conditional independence assumption foundational to Naive Bayes, artificially inflating likelihood estimates and corrupting the calibrated posterior probabilities. Therefore, PRISM and the Bayesian Network were restricted to a strictly complementary, compact subset of purely patient-level metadata (Age, Gender, Lesion region, and Skin tone).

Table 3 summarizes the cross-validation results under these specific configurations. The Wald omnibus test revealed significant differences among the evaluated methods across all classification metrics (all $p < 0.001$). Following this, post-hoc pairwise comparisons demonstrated that PRISM achieved significantly higher balanced accuracy than the vision-only baseline ($p = 0.049$). When compared to probabilistic methods, PRISM significantly outperformed the Bayesian Network ($p < 0.001$), which operated on the exact same clinical feature set. Remarkably, despite utilizing a restricted feature set compared to the deep learning fusion baselines, PRISM also achieved significantly higher balanced accuracy than MetaBlock-SE ($p < 0.001$) and Cross-Attention ($p = 0.022$), while demonstrating performance statistically comparable to the more parameter-intensive Cross Modality Fusion ($p = 0.079$).

Interestingly, our statistical analysis revealed a behavioral divergence in the hierarchical Bayesian Network. While it achieved significantly higher overall precision than PRISM ($p < 0.001$), this came at a severe expense to balanced accuracy ($p < 0.001$). This tradeoff indicates that in highly heterogeneous and severely imbalanced datasets like MILK10k, attempting to learn complex joint distributions induces a strong, conservative prediction bias. The model defaults to predicting only the most heavily represented classes, artificially inflating precision while failing to identify minority clinical presentations.

**Table 3:** Classification performance on the MILK10k dataset. The proposed PRISM framework is compared against vision-only baselines (No Metadata) and state-of-the-art multimodal fusion methods across four vision backbones. Values are reported as mean ± standard deviation.

Model	BACC	F1-Score	AUC	Specificity	Precision
No Metadata					
DaViT-Tiny	$0.530\pm 0.024$	$0.425\pm 0.017$	$0.901\pm 0.018$	$0.960\pm 0.002$	$0.397\pm 0.017$
EfficientNet-B0	$0.496\pm 0.010$	$0.411\pm 0.009$	$0.867\pm 0.020$	$0.958\pm 0.001$	$0.389\pm 0.007$
MobileNet-V3	$0.451\pm 0.020$	$0.365\pm 0.011$	$0.846\pm 0.013$	$0.954\pm 0.001$	$0.348\pm 0.006$
SwinV2-Tiny	$0.483\pm 0.042$	$0.388\pm 0.059$	$0.882\pm 0.014$	$0.955\pm 0.006$	$0.371\pm 0.060$
PRISM (ours)					
DaViT-Tiny	$0.546\pm 0.032$	$0.428\pm 0.025$	$0.903\pm 0.017$	$0.959\pm 0.003$	$0.405\pm 0.023$
EfficientNet-B0	$0.535\pm 0.045$	$0.403\pm 0.024$	$0.878\pm 0.014$	$0.954\pm 0.003$	$0.395\pm 0.018$
MobileNet-V3	$0.463\pm 0.017$	$0.361\pm 0.016$	$0.870\pm 0.012$	$0.951\pm 0.001$	$0.355\pm 0.014$
SwinV2-Tiny	$0.512\pm 0.044$	$0.404\pm 0.060$	$0.890\pm 0.022$	$0.955\pm 0.005$	$0.388\pm 0.057$
Cross-Attention					
DaViT-Tiny	$0.506\pm 0.034$	$0.400\pm 0.043$	$0.879\pm 0.034$	$0.955\pm 0.005$	$0.381\pm 0.044$
EfficientNet-B0	$0.463\pm 0.029$	$0.372\pm 0.034$	$0.876\pm 0.012$	$0.952\pm 0.004$	$0.354\pm 0.031$
MobileNet-V3	$0.462\pm 0.030$	$0.355\pm 0.017$	$0.866\pm 0.014$	$0.950\pm 0.002$	$0.341\pm 0.012$
SwinV2-Tiny	$0.512\pm 0.052$	$0.395\pm 0.017$	$0.882\pm 0.008$	$0.954\pm 0.001$	$0.384\pm 0.025$
Cross Modality Fusion					
DaViT-Tiny	$0.518\pm 0.036$	$0.420\pm 0.036$	$0.893\pm 0.019$	$0.959\pm 0.003$	$0.398\pm 0.029$
EfficientNet-B0	$0.472\pm 0.018$	$0.383\pm 0.012$	$0.875\pm 0.014$	$0.955\pm 0.002$	$0.362\pm 0.011$
MobileNet-V3	$0.479\pm 0.029$	$0.379\pm 0.013$	$0.871\pm 0.006$	$0.953\pm 0.001$	$0.360\pm 0.009$
SwinV2-Tiny	$0.512\pm 0.016$	$0.433\pm 0.037$	$0.892\pm 0.015$	$0.959\pm 0.006$	$0.415\pm 0.027$
Bayesian Network					
DaViT-Tiny	$0.407\pm 0.059$	$0.431\pm 0.053$	$0.901\pm 0.009$	$0.959\pm 0.005$	$0.544\pm 0.060$
EfficientNet-B0	$0.349\pm 0.029$	$0.386\pm 0.031$	$0.877\pm 0.011$	$0.955\pm 0.001$	$0.580\pm 0.065$
MobileNet-V3	$0.319\pm 0.030$	$0.343\pm 0.036$	$0.868\pm 0.011$	$0.952\pm 0.002$	$0.507\pm 0.083$
SwinV2-Tiny	$0.386\pm 0.077$	$0.408\pm 0.067$	$0.889\pm 0.021$	$0.956\pm 0.006$	$0.497\pm 0.034$
MetaBlock-SE					
DaViT-Tiny	$0.410\pm 0.036$	$0.336\pm 0.022$	$0.821\pm 0.030$	$0.949\pm 0.002$	$0.328\pm 0.022$
EfficientNet-B0	$0.340\pm 0.054$	$0.249\pm 0.053$	$0.766\pm 0.033$	$0.935\pm 0.005$	$0.264\pm 0.030$
MobileNet-V3	$0.336\pm 0.019$	$0.248\pm 0.036$	$0.786\pm 0.032$	$0.936\pm 0.005$	$0.263\pm 0.021$
SwinV2-Tiny	$0.394\pm 0.036$	$0.310\pm 0.034$	$0.801\pm 0.021$	$0.943\pm 0.004$	$0.304\pm 0.025$

**Fig. 4:**
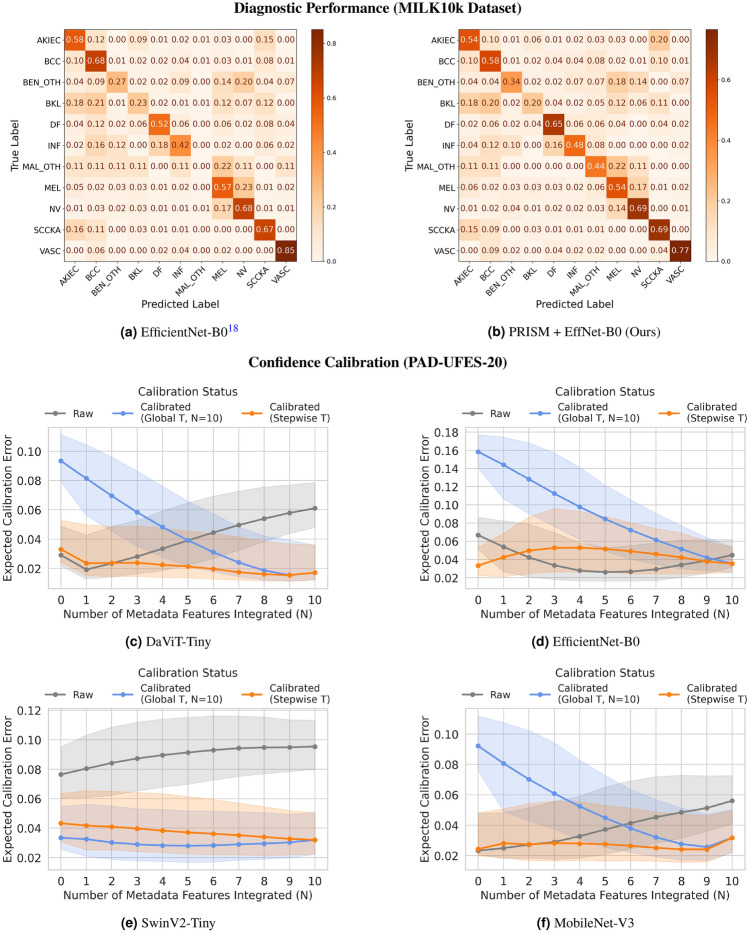
Top: MILK10k confusion matrices for (**a**) EfficientNet-B0 baseline and (**b**) PRISM (ours). PRISM corrects the baseline’s failure on the rare MAL_OTH class. Bottom: Out-of-fold Expected Calibration Error (ECE) trajectories across four architectures on PAD-UFES-20. The Global Temperature degrades calibration when few clinical data is used. Conversely, our Stepwise Calibration maintains probabilistic alignment across all stages of feature integration (*N*). Shaded areas denote 95% confidence intervals calculated with Bootstrapping with 10,000 resamples.

Furthermore, Fig. 4a–b present the confusion matrices for the EfficientNet-B0 (standalone and with PRISM’s metadata integration) whereas Supplementary Figure S6 displays the results for the Siamese ResNet-50^36^ baseline. To ensure a fair comparison, it is important to note the similarities and differences in the training protocols. Both the baseline and our proposed method utilized a 5-fold cross-validation strategy stratified by target classes, and we ensured exact comparability by employing the same random seed to replicate the baseline’s specific data splits. Furthermore, both implementations explicitly addressed the severe class imbalance by employing a Cross-Entropy loss function weighted by inverse class frequencies. The primary protocol differences lie in the optimization strategy–where PRISM utilized the Adam optimizer with a 1cycle learning rate policy, compared to the baseline’s AdamW with a step-down schedule and a 0.01 label smoothing penalty–and the specific input requirements. To assess the stability of these results across the identical folds, 95% confidence intervals (CIs) were computed analytically using the Student’s t-distribution.

The Siamese ResNet-50 requires paired visual inputs of both clinical and dermoscopic images to achieve a mean recall of 0.426 (95% CI: 0.392–0.459)^36^. In contrast, despite operating under identical cross-validation and loss-weighting constraints for imbalance, PRISM demonstrates superior sensitivity by leveraging a single dermoscopic image integrated with patient clinical metadata. Even while employing the more compact EfficientNet-B0 backbone instead of a computationally heavy dual-stream ResNet-50, PRISM achieved a higher recall of 0.535 (95% CI: 0.479–0.591) while maintaining a competitive specificity of 0.954 (95% CI: 0.950–0.958) against the baseline’s 0.960 (95% CI: 0.959–0.961)^36^.

### Analysis of confidence calibration and probabilistic reliability

While the integration of clinical metadata via the PRISM framework significantly improves diagnostic sensitivity by treating the vision model’s softmax outputs as Bayesian priors, the raw Naive Bayes multiplication dynamically shifts the resulting confidence distributions. To ensure the framework’s outputs represent statistically reliable confidence estimates at every step of the diagnostic trajectory, we implemented a comprehensive calibration and evaluation protocol.

#### Quantitative assessment protocol

Model calibration was quantitatively assessed using the Expected Calibration Error (ECE)^46,47^, which computes the weighted average of the absolute difference between accuracy and confidence across 10 uniformly spaced bins. To provide an unbiased assessment of the models’ generalization capabilities, all reported ECE values and corresponding reliability diagrams were computed using the pooled out-of-fold predictions from our cross-validation pipeline. Furthermore, we report the $95\%$ Confidence Interval (CI) for every ECE value, estimated via bootstrapping with 10,000 resamples^48^.

#### Dataset-dependent confidence shifts

Comparing the out-of-fold vision baselines against the raw, uncalibrated PRISM outputs revealed that the metadata’s impact on confidence calibration is highly dataset-dependent. On the PAD-UFES-20+ dataset (Supplementary Figures S7–S8), the vision baselines were generally well-calibrated (e.g., DaViT-Tiny baseline ECE = 0.025, 95% CI: 0.019–0.031). However, integrating clinical metadata inherently induced overconfidence, consistently elevating the ECE across all backbones to a range of 0.052 to 0.066. Conversely, on the highly imbalanced MILK10k dataset (Supplementary Figures S9–S10), the clinical metadata frequently acted as a calibrating anchor. Raw metadata integration dramatically reduced the ECE for initially poorly calibrated vision models, dropping the EfficientNet-B0 ECE from 0.153 to 0.075, the MobileNet-V3 ECE from 0.110 to 0.045, and achieving near-perfect calibration for the DaViT-Tiny architecture (baseline ECE = 0.058 improving to PRISM ECE = 0.011, 95% CI: 0.011–0.028). Because the metadata’s raw impact on probabilistic alignment fluctuates, a standardized calibration methodology is strictly required.

#### Stepwise post-hoc calibration

To correct miscalibrations and restore confidence alignment for models trained on the PAD-UFES-20 dataset, we employed post-hoc Temperature Scaling^46^. To calibrate these models, we utilized a strictly independent hold-out set consisting of samples from the expanded PAD-UFES-20+ dataset (clinical data collected from 2021 onwards). This temporal split ensures that the calibration data is entirely isolated from the original PAD-UFES-20 training and validation distributions.

Because our evaluation relies on cross-validation, the calibration strategy was applied systematically across every fold. For each cross-validation fold, the specific trained model checkpoint was evaluated on the independent temporal hold-out set to extract uncalibrated logits. We then optimized a scalar temperature parameter *T* by minimizing the Cross-Entropy loss on these hold-out logits using the L-BFGS algorithm^49^, trained for up to 100 epochs with a learning rate of 0.01. To mathematically guarantee $T> 0$, the scaling factor was parameterized as $\log (T)$. Once the optimal *T* was learned for a given fold, it was applied to scale the uncalibrated validation logits for that specific fold. Finally, these calibrated predictions were aggregated across all folds to calculate the pooled out-of-fold ECE.

The standard application of this method yields a single, global temperature (*T*) per fold, learned on the complete metadata profile (10 features). However, because the Naive Bayes framework assumes conditional independence, the multiplication of sequential likelihoods inherently compounds overconfidence as the volume of evidence increases. Consequently, a global *T* scaled for the maximum evidence mathematically over-penalizes predictions made with fewer clinical features.

To systematically evaluate this effect, we computed predictions for all 1,024 ($2^{10}$) possible metadata combinations for every sample. We achieved this by artificially masking any clinical variable not included in a given combination. Importantly, we never removed samples from the evaluation cohort of any specific combination to artificially construct “complete” records. For example, if a specific combination of size $N=2$ permitted the model to query only age and anatomical site, we masked the remaining eight variables. If a patient’s real-world record was naturally missing the anatomical site, we did not discard their data; the model simply evaluated them using only their age. Therefore, the feature count (*N*) represents the number of clinical questions the model is allowed to ask, rather than the strict number of non-empty answers a patient possesses. By keeping the total pool of evaluated lesions constant across all combinations–from the vision-only baseline ($N=0$) to the fully integrated protocol ($N=10$)–we prevented severe selection bias, ensuring our calibration metrics accurately reflect real-world clinical deployment. For each feature count ($N \in \{0, \dots , 10\}$), we aggregated the out-of-fold predictions across all combinations of that specific size to calculate the mean ECE. To capture both sample-level variance and combinatorial uncertainty, the 95% Confidence Intervals (CIs) were estimated using 10,000 bootstrap resamples. In each iteration for a given *N*, we drew a random subset of instances ($n=2,298$) with replacement, and evaluated their ECE using a single, randomly chosen feature combination of size *N*. At the boundaries ($N=0$ and $N=10$), where only one feature combination exists, this method naturally defaults to measuring sample-level predictive uncertainty.

Our combinatorial evaluation (Fig. 4c–f) reveals that applying a global *T* to early diagnostic steps may severely degrade probabilistic alignment. For example, when evaluating the vision-only prior ($N=0$ features) using the DaViT-Tiny backbone, the global *T* artificially suppresses confidence, resulting in a highly miscalibrated ECE of 0.093 (95% CI: 0.079–0.111). This degradation is even more severe for EfficientNet-B0, where the global *T* drives the vision-only ECE to 0.158 (95% CI: 0.140–0.177).

To resolve this combinatorial penalty mismatch, we implemented a Stepwise Calibration protocol. Instead of optimizing a single global scaling factor, we first aggregated the uncalibrated temporal hold-out predictions across all possible feature combinations of a specific size *N* ($N \in \{0, \dots , 10\}$). We then utilized the L-BFGS algorithm on this aggregated combinatorial data to learn an independent temperature ($T_N$) per fold for each exact volume of available evidence. This approach dynamically pairs the severity of the temperature penalty with the amount of clinical metadata integrated. As illustrated in Supplementary Figure S11, the learned optimal temperature $T_N$ monotonically increases as a function of *N* across all evaluated backbones. This empirically validates the theoretical limitation of the Naive Bayes conditional independence assumption: as more clinical features are multiplied, the resulting posterior probabilities become sharper, demanding a proportionately larger penalty to restore calibration.

As demonstrated in Fig. 4c and f, Stepwise Calibration consistently preserves probabilistic alignment for well-calibrated baselines like DaViT-Tiny and MobileNet-V3. For DaViT-Tiny, it restores the vision-only ECE to a highly calibrated 0.033 (95% CI: 0.024–0.053) while maintaining a fully-integrated ($N=10$) ECE of 0.017 (95% CI: 0.012–0.036).

Importantly, our combinatorial analysis also highlights architecture-dependent calibration dynamics. As seen in Fig. 4d, for models like EfficientNet-B0, the inherent compounding effect of Naive Bayes occasionally acts as a natural confidence sharpener, causing the uncalibrated ECE to temporarily drop lower than the calibrated bounds during intermediate steps (reaching 0.026 at $N=5$). Similarly, as seen in Fig. 4e, for highly miscalibrated baselines like SwinV2-Tiny (uncalibrated ECE > 0.07), the heavy penalty of the global *T* acts as a strong correction that performs on par with stepwise scaling. Nevertheless, Stepwise Calibration remains a strict methodological requirement: it acts as a safety bound that prevents the catastrophic early-step failures induced by global scaling, allowing every intermediate probability distribution presented in the PRISM audit to remain clinically reliable.

### Interpretability analysis

A defining advantage of PRISM lies in its capacity to disentangle the decision-making process–a feature absent in conventional deep fusion architectures. While standard approaches mask the specific contribution of clinical variables, our framework explicitly quantifies the impact of each clinical attribute, shifting the focus from simply what the model predicts to why. By inspecting the stepwise evolution of diagnostic probabilities as individual metadata features are integrated, we establish a transparent audit trail of the model’s reasoning, offering clinicians a level of interpretability not inherently available in end-to-end models.

To demonstrate this, we present four illustrative cases from the validation set of the PRISM (DaViT-Tiny) model. Rather than employing random sampling, we purposively selected these examples to provide a balanced representation of the stepwise integration mechanism across diverse clinical scenarios. Three cases showcase successful multimodal integration where metadata corrects an initial visual misclassification (a Melanoma misclassified as Nevus, a Basal Cell Carcinoma as Actinic Keratosis, and a Seborrheic Keratosis as Nevus). Conversely, to ensure a comprehensive and transparent evaluation of the model’s limitations, we explicitly included one instance where the metadata incorrectly overrides a valid visual prediction (a Nevus misclassified as Melanoma). This deliberate selection demonstrates that the model’s interpretability is equally valuable for conducting failure analysis and understanding the boundaries of the metadata prior. Furthermore, to ensure the visualized stepwise updates accurately reflect true diagnostic confidence, all probabilities presented in these analyses correspond to the post-hoc calibrated estimates obtained via our Stepwise Temperature Scaling strategy.

#### Case 1: Melanoma vs Nevus

**Fig. 5:**
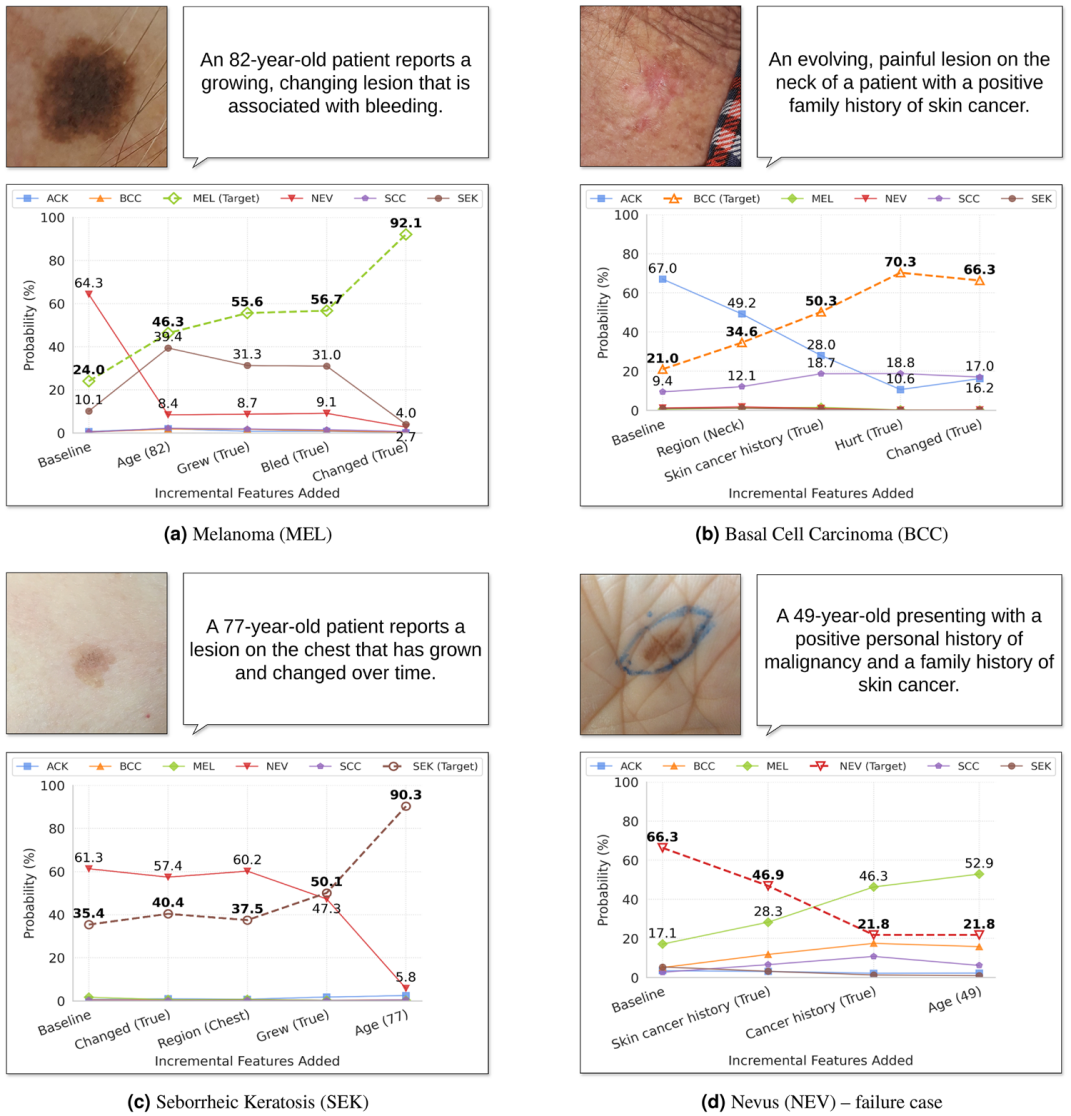
Interpretability case study for a Melanoma (MEL) lesion. The vision-only model (Baseline) incorrectly predicts Nevus (NEV, 64.3%). The addition of Age (82) corrects the primary diagnosis, and the Changed (True) feature solidifies the model’s confidence to 92.1% MEL. Interpretability case study for a Basal Cell Carcinoma (BCC). The baseline model misidentifies the lesion as Actinic Keratosis (ACK, 67.0%). The Skin cancer history (True) feature inverts the diagnosis, and Hurt (True) reinforces the correct BCC prediction to 70.3%. Interpretability case study for a Seborrheic Keratosis (SEK). The vision model incorrectly predicts Nevus (NEV, 61.3%). While Grew (True) inverts the diagnosis, the Age (77) feature provides the decisive clinical context, increasing the SEK probability to 90.3%. Failure analysis case study for a benign Nevus (NEV). The vision-only model is initially correct (66.3% NEV). However, a combination of misleading red flag metadata (Skin cancer history (True), Cancer history (True)) compounds to override the correct prediction, resulting in a final misdiagnosis of Melanoma.

Figure 5a details the probabilistic evolution for a Melanoma (MEL) lesion that was initially misdiagnosed by the vision-only model. At the Baseline, the model confidently misclassified the lesion as a benign Nevus (NEV) with 64.3% probability—a clinically realistic error given the visual similarity—while the ground truth MEL was a distant second at 24.0%. The introduction of the first metadata feature, Age (82), immediately shifted the diagnosis: the NEV probability dropped to 8.4%, while the probabilities for MEL (46.3%) and Seborrheic Keratosis (SEK, 39.4%) rose sharply, refining the differential diagnosis to a more age-appropriate one (MEL vs. SEK). Subsequently, the addition of the symptom Grew (True) further increased the suspicion of MEL to 55.6% while decreasing confidence in the typically stable SEK. The Bled (True) information, however, offered little diagnostic impact. Finally, the inclusion of Changed (True)—an indicator corresponding to ’Evolving’ in the ABCDEs^50^—resolved the ambiguity, causing the MEL confidence to leap to 92.1% as the SEK probability collapsed to 4.0%.

This step-by-step visualization moves beyond a single, black box output. It provides an interpretable audit trail, allowing a physician to see which metadata features were most influential and how they were used to refine the diagnosis. This transparency is important for clinical adoption, as it allows the end-user to validate the model’s decision-making process against their own domain expertise.

#### Case 2: Basal Cell Carcinoma vs Actinic Keratosis

A different clinical ambiguity is explored in Fig. 5b, involving a Basal Cell Carcinoma (BCC) lesion misidentified as Actinic Keratosis (ACK). This scenario represents a common challenge, as both lesions are non-pigmented and commonly appear in sun-exposed areas. The vision-only model’s baseline assessment assigned a high 67.0% probability to ACK, while the ground truth BCC received only 21.0%. The first feature, Region (Neck), was insufficient to correct the misdiagnosis, only slightly reducing the ACK probability to 49.2% while increasing BCC to 34.6%. The decisive feature was Skin cancer history (True). This information, an intrinsic risk factor for BCC^51^, inverted the probabilities, elevating BCC to the most likely diagnosis (50.3%) and dropping ACK to 28.0%. The subsequent inclusion of the symptom Hurt (True) further increased the BCC confidence to 70.3% while the ACK probability was reduced to 10.6%. The final feature, Changed (True), caused a minor downward fluctuation, but the model’s correct prediction of BCC remained at 66.3%. This case demonstrates the model’s ability to resolve a common diagnostic ambiguity (BCC vs. ACK) by weighing risk factors and symptoms.

#### Case 3: Seborrheic Keratosis vs Nevus

To further demonstrate the model’s ability to resolve ambiguity, we analyze a Seborrheic Keratosis (SEK) that was visually mistaken for a Nevus (NEV), as shown in Fig. 5c. The baseline probability reflects this visual confusion, with the model assigning 61.3% to the incorrect NEV diagnosis, while the ground truth SEK received only 35.4%. The introduction of Changed (True) and Region (Chest) caused only minor fluctuations, with the incorrect NEV diagnosis holding. The first pronounced shift occurred with the feature Grew (True). This symptom, which can be associated with SEK^52^, inverted the probabilities, elevating SEK to the most likely diagnosis (50.1%) and dropping NEV to 47.3%. The final feature, Age (77), provided the decisive clinical context. As SEKs are strongly correlated with advanced age while new nevi are not, this information resolved the ambiguity. The probability for SEK changed to 90.3%, while the probability for NEV was reduced to 5.8%. This case demonstrates the model’s ability to use demographic data to override an incorrect visual-only prediction.

#### Case 4: Nevus vs Melanoma

Finally, Fig. 5d illustrates the selected failure case, demonstrating how misleading metadata can override a correct visual-only prediction. The lesion is a benign Nevus (NEV), which the vision-only model correctly identified at the Baseline with high confidence (66.3%), while Melanoma (MEL) was a distant possibility at 17.1%. The introduction of Skin cancer history (True)–a clinically relevant but non-specific risk factor–immediately introduced ambiguity, dropping the NEV probability to 46.9% and raising MEL to 28.3%. The inversion occurred with the addition of a second, separate feature: Cancer history (True). This general cancer history, compounded with the specific skin cancer history, caused the model to incorrectly favor MEL (46.3%) over the ground truth NEV (21.8%). The final feature, Age (49), did not provide corrective information and further solidified this misdiagnosis, leading to a final incorrect prediction of MEL with 52.9% confidence. This case demonstrates how a combination of melanoma risk factors^51^ can compound to mislead the classifier, overriding a correct baseline visual assessment.

Importantly, this individual failure case must be contextualized within the broader statistical trade-offs inherent to the PRISM framework. To map these dynamics, we quantified the rates of metadata-driven corrections and spurious inversions across all out-of-fold validation sets, detailed in our global transition matrices (Supplementary Tables S6–S8) and per-class inversion diagrams (Supplementary Figures S12–S14). On the PAD-UFES-20 and PAD-UFES-20+ datasets, metadata integration yielded both an increase in balanced accuracy and a strong positive net improvement in absolute predictions. For example, on the PAD-UFES-20 dataset, PRISM successfully corrected between 13.66% and 18.45% of vision-only errors while only inverting 4.92% to 8.09% of initially correct predictions. However, the MILK10k dataset explicitly contextualizes the limitations highlighted by our failure case. On this dataset, while PRISM still successfully increased the balanced accuracy across all architectures (e.g., lifting EfficientNet-B0 from 0.496 to 0.535), the global transition analysis revealed a net negative improvement in absolute predictions for most models. Specifically, the absolute inversion rates (e.g., 10.76% for EfficientNet-B0) outpaced the correction rates (5.67%). This divergence–where overall BACC improves despite a net increase in absolute misclassifications–occurs because the metadata effectively corrects predictions in difficult, underrepresented minority classes, which heavily pulls up the balanced accuracy. However, this comes at the cost of over-predicting those risk factors in the much larger majority classes, driving up the absolute number of inversions. Therefore, while compounded clinical risk factors can occasionally overpower visual evidence to yield false positives, this probabilistic shifting is the exact mechanism that enables the framework to achieve higher minority-class sensitivity and better overall disease capture.

## Discussion

We introduced PRISM, a probabilistic framework that enhances interpretability by establishing a clear diagnostic trail, exemplified in Fig. 5a–d. Unlike end-to-end deep fusion architectures that often mask the specific contributions of visual and clinical data, our approach utilizes a probabilistic framework to render the decision-making process explicit. By treating the deep learning vision prediction as an initial prior and sequentially updating it with patient information, our method aligns with the standard dermatological practice of validating visual impressions with patient history, and allows the influence of every feature to be individually evaluated. Beyond its inherent interpretability, across all datasets, PRISM demonstrates competitive, and often superior performance compared with both the vision-only baselines and state-of-the-art fusion methods such as Cross Attention, Cross Modality Fusion, Bayesian Network and MetaBlock-SE.

On the PAD-UFES-20 dataset (Table 1), PRISM consistently outperforms all competing methods in balanced accuracy across all vision backbones (all adjusted $p < 0.001$). In the more complex PAD-UFES-20+ dataset (Table 2), our method maintains competitive performance, achieving a balanced accuracy of $0.698 \pm 0.012$ and AUC of $0.937 \pm 0.005$ for DaViT-Tiny, which is on par with Cross Attention ($p_{bacc} = 0.604$, $p_{auc} = 0.604$) and Cross Modality Fusion ($p_{bacc} = 0.324$, $p_{auc} = 0.604$). Notably, the probabilistic model achieves similar discriminative ability while using a simpler, interpretable mechanism, and a radically reduced number of parameters, as seen in Fig. 3b.

A similar pattern emerges in MILK10k (Table 3), a larger and more heterogeneous dataset. Here, PRISM successfully yields a statistically significant improvement over the vision-only baseline in balanced accuracy ($p = 0.049$), achieving a BACC of $0.546 \pm 0.032$ with the DaViT-Tiny backbone. The primary benefit is concentrated in the challenging MAL_OTH class, which suffers from low sample count and clinical overlap with other malignant classes. As shown in Fig. 4a–b, our approach boosts the BACC for this class from 0% to 44%. Moreover, PRISM’s metadata integration increased the total percentage of MAL_OTH samples identified as any malignant class from 33% to 77%. Specifically, while the baseline model classified these samples as BCC (11%), MEL (22%), and MAL_OTH (0%), PRISM successfully reassigned an additional 44% of the samples to the correct MAL_OTH class without reducing the BCC (11%) and MEL (22%) true positive rates. Although the overall use of metadata caused a slight drop in sensitivity for the true BCC class–primarily because some actual BCC lesions were reclassified as MAL_OTH–this confusion between malignant subtypes has minimal clinical impact, as both diagnoses ultimately require surgical excision.

Furthermore, a key advantage of the proposed method lies in its capacity to address a persistent challenge in clinical decision support systems: the inability to isolate and quantify the specific contribution of each clinical variable to the final diagnosis. Clinicians frequently report that black box fusion methods masks the reasoning process, making it impossible to discern whether a prediction was driven by visual features or specific risk factors. PRISM addresses this by enabling a direct, granular assessment of how each attribute shifts the diagnostic probability. Because our model remains robust even when clinical information is partially missing (Fig. 3a and Supplementary Figure S5), it allows for a dynamic evaluation where predictions evolve smoothly as metadata are incrementally introduced. This creates a transparent diagnostic audit trail, answering the question of how much a specific piece of information–such as age or anatomical region–weighs in the decision, thereby strengthening clinical trust. Importantly, for these probabilistic audit trails to be clinically actionable, the underlying confidence estimates must remain sufficiently reliable at every intermediate step. As demonstrated by our calibration analysis, the inherent compounding overconfidence of the Naive Bayes assumption would otherwise artificially inflate these early-step predictions. By dynamically applying our Stepwise Calibration protocol to scale the temperature penalty to the exact volume of integrated evidence, we provide a necessary corrective bound. While intermediate probability distributions may still exhibit minor calibration fluctuations, this stepwise approach prevents severe early-step overconfidence, ensuring that each incremental update remains statistically stable. This allows PRISM to offer much more reliable confidence estimates throughout the reasoning process–ultimately reducing the fully-integrated Expected Calibration Error to 0.017 for the DaViT-Tiny backbone–without sacrificing the diagnostic sensitivity gained from the metadata.

The case studies presented in Fig. 5a–d further substantiate the interpretability and clinical relevance of the proposed framework. In the successful examples, the progressive inclusion of metadata corrected initial visual misclassifications by realigning diagnostic probabilities according to clinically meaningful cues such as age, lesion evolution, and patient history. While this method does not fully resolve the interpretation challenges inherent to the visual backbone itself, it offers a significant contribution toward transparent data integration. This behavior demonstrates that the model adjusts its diagnosis process in a manner consistent with established dermatological logic. Conversely, the failure case–where metadata led to an incorrect prediction–highlights the transparency of the approach, as it exposes how specific risk-related attributes can bias the inference process.

Overall, these results validate that PRISM delivers on two primary contributions required for real-world deployment: enhanced interpretability and robustness to missing data. Unlike opaque attention-based fusion methods, our probabilistic approach maintains stability regardless of data availability and provides a clear diagnostic audit trail. These features make PRISM well-suited for clinical decision-making, where clinical data is heterogeneous and the reasoning behind a diagnosis is a prerequisite for clinical adoption.

## Limitations

A limitation of the current PRISM framework is its inherent assumption that clinical metadata is missing at random. By handling missing values through omission–uniformly setting their likelihoods to 1 during the stepwise update–the model ignores any potential predictive signal encoded in the missingness itself. As is common in clinical practice, data is often Missing Not At Random (MNAR); for instance, a physician might be less likely to record a comprehensive symptom history for a lesion that is visibly benign. Consequently, PRISM will not exploit the absence of this data as a signal for a benign prediction, potentially sacrificing some predictive accuracy compared to end-to-end models that explicitly encode missing states. However, this conservative approach is a deliberate design choice prioritizing clinical safety. Exploiting informative missingness is highly susceptible to learning spurious hospital workflows or administrative biases rather than true pathology. By ensuring that predictions are only updated by explicitly observed clinical evidence, PRISM avoids the dangerous pitfall of predicting a benign outcome simply because a clinician was rushed and omitted a metadata field.

A further theoretical limitation of our framework stems from the underlying Naive Bayes assumption of conditional independence. Because clinical metadata features are frequently correlated in practice, the multiplicative update mechanism can effectively “double-count” redundant evidence. As demonstrated in our probabilistic reliability analysis, this often leads to overconfident posterior estimates, pushing the raw output distributions toward extreme values of 0 or 1. Consequently, the raw, uncalibrated posterior probabilities generated by the Naive Bayes step should not be directly interpreted as fully calibrated clinical risk scores. To safely utilize these outputs as reliable measures of true diagnostic confidence, it is strictly necessary to apply rigorous post-hoc calibration techniques–such as the Stepwise Temperature Scaling demonstrated in our methodology. Furthermore, it is important to note that the efficacy of this post-hoc calibration was validated on clinical scenarios integrating a moderate number of features (up to 10). Scaling this framework to environments with dozens of highly correlated variables may compound the independence violations to a degree that requires more complex calibration interventions.

A final practical limitation of the current PRISM implementation–and indeed of the standard end-to-end multimodal baselines evaluated in this study–sis the absence of an explicit out-of-distribution detection mechanism. Because the vision backbone operates as a standard closed-set softmax classifier, it is mathematically forced to assign any input image, including non-dermatological or entirely out-of-domain photos, to one of the predefined skin lesion classes. While the primary scope of this work is establishing a robust and interpretable multimodal fusion framework, we acknowledge that for safe, clinical-facing deployment, this framework cannot operate in isolation. Future real-world implementations must be preceded by an input-domain safeguard, thereby preventing the presentation of misleading diagnoses.

## Conclusion

In this work, we introduced PRISM, a probabilistic framework designed to bridge the gap between predictive accuracy and clinical interpretability in skin cancer diagnosis. By modeling the integration of deep vision models and patient metadata as a stepwise Bayesian update, our approach replicates the cognitive workflow of dermatological assessment. Validated across three diverse datasets, PRISM demonstrated superior stability, consistently outperforming vision-only baselines while exhibiting inherent robustness to incomplete patient records, a scenario where attention-driven deep fusion models often failed. Beyond quantitative performance, the framework’s primary contribution is the establishment of a transparent diagnostic audit trail. To ensure these incremental updates are clinically actionable, we introduced a Stepwise Calibration protocol. By dynamically scaling the temperature penalty to the exact volume of integrated evidence, this approach mitigates the compounding overconfidence inherent in sequential Bayesian updating, maintaining statistically stable confidence estimates at every intermediate step. By enabling the granular evaluation of clinical evidence backed by reliable probability distributions, PRISM aligns computer-aided diagnosis with medical reasoning, offering a robust and trustworthy tool for real-world clinical decision support.

Future work will focus on extending the underlying graphical model to capture even more nuances of the dermatological workflow. One promising direction is the integration of multi-view analysis. Just as a dermatologist examines a lesion from multiple angles or lighting conditions, PRISM could be adapted to aggregate probabilistic evidence from multiple images of the same lesion, thereby enhancing diagnostic confidence. Finally, we aim to leverage the framework’s modularity to integrate heterogeneous imaging modalities. By consolidating macroscopic clinical photography and dermoscopic detail into a unified vision-based prior, PRISM can formulate a comprehensive visual assessment, effectively replicating the multi-modal examination performed by dermatologists.

## Supplementary Information

Supplementary Information.

## Data Availability

The PAD-UFES-20 dataset analyzed during the current study is publicly available in the Mendeley Data repository at https://data.mendeley.com/datasets/zr7vgbcyr2/1 The MILK10k dataset is publicly available at https://challenge.isic-archive.com/landing/milk10k/ Regarding the PAD-UFES-20+ dataset, due to patient privacy protections and the ongoing nature of the data collection, the raw images and full metadata cannot be made publicly available at this time. However, the full dataset is scheduled for public release in 2026. In the interim, de-identified data may be available from the corresponding author on reasonable request. The source code to reproduce the experiments is publicly available on GitHub at https://github.com/life-ufes/prism A live inference demo is hosted on Hugging Face Spaces at https://huggingface.co/spaces/pedrobouzon/prism
