# Supplementary material for: PRISM: a clinically interpretable stepwise framework for multimodal skin cancer diagnosis
Source: Sci Rep. 2026 May 21;16:23262. doi: 10.1038/s41598-026-47756-4 (PMC13402622; doi:10.1038/s41598-026-47756-4)
Supplement: Supplementary file 1 — Supplementary Information. [file 41598_2026_47756_MOESM1_ESM.pdf]

Supplementary Figures

Figure S1: Comparison of data completeness between the PAD-UFES-20 and PAD-UFES-20+ datasets.

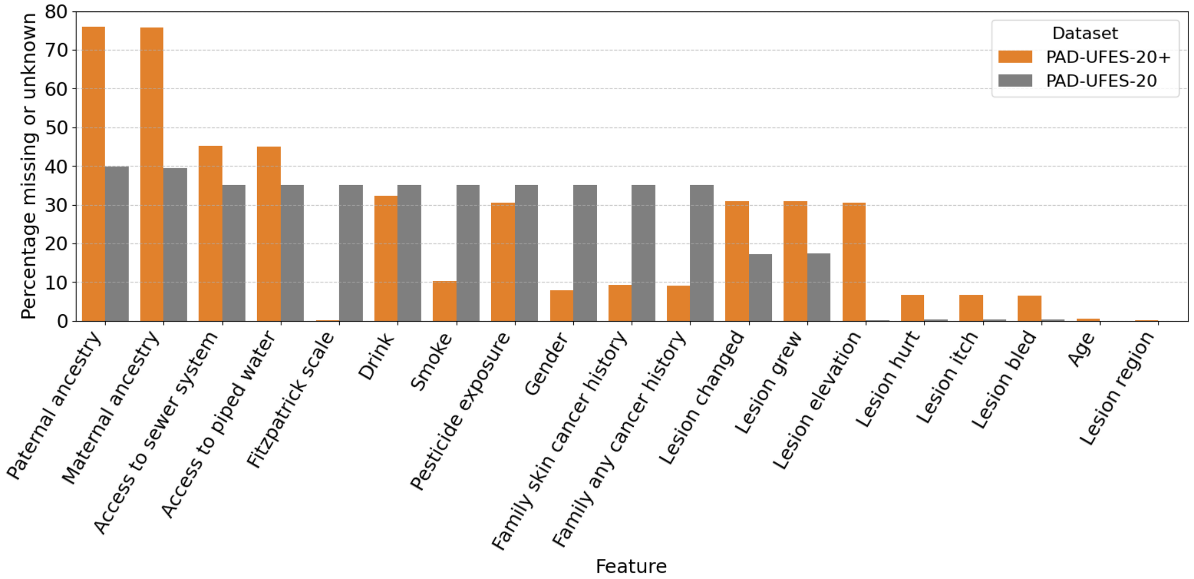

The bar chart displays the percentage of missing or unknown entries for each clinical feature. The proposed PAD-UFES-20+ dataset (orange) is compared against the original PAD-UFES-20 dataset (gray), highlighting variations in metadata availability across the features.

Figure S2: Diagnostic diversity on the PAD-UFES-20 dataset[1]. Representative smartphone-captured clinical images are shown for each of the six evaluated classes: (a) Actinic keratosis (ACK), (b) Basal cell carcinoma (BCC), (c) Squamous cell carcinoma (SCC), (d) Seborrheic keratosis (SEK), (e) Nevus (NEV), and (f) Melanoma (MEL).

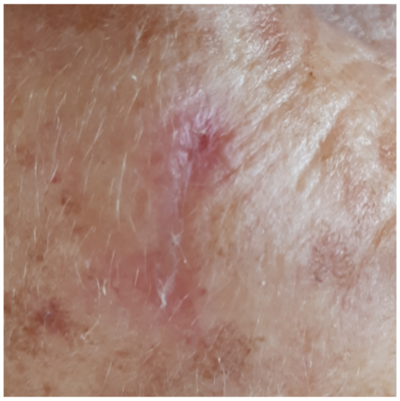

(a) Actinic Keratosis (ACK)

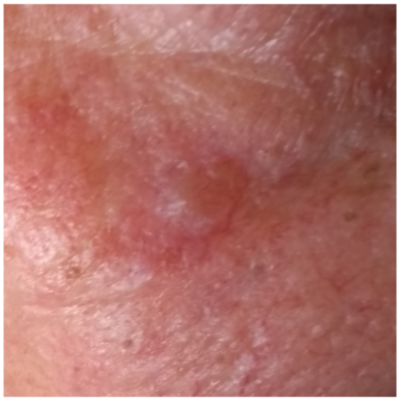

(b) Basal Cell Carcinoma (BCC)

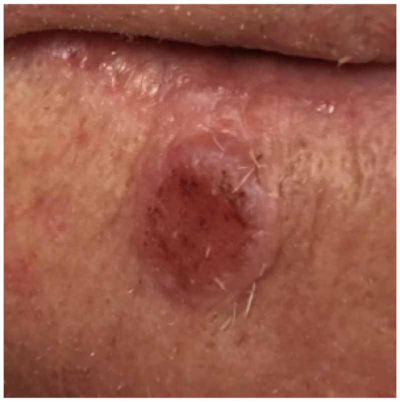

(c) Squamous Cell Carcinoma (SCC)

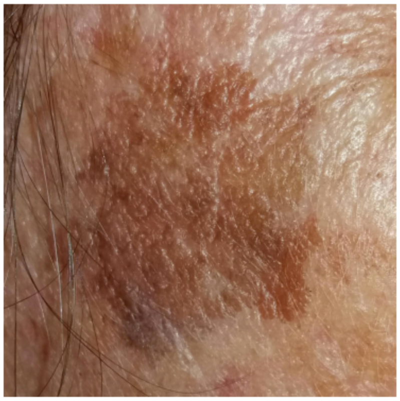

(d) Seborrheic Keratosis (SEK)

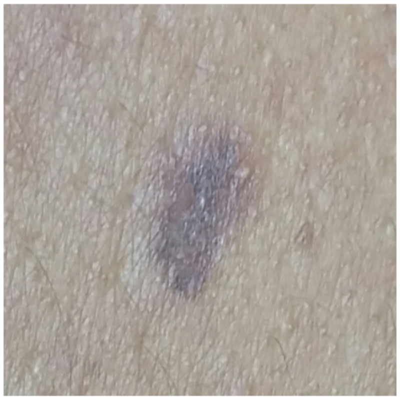

(e) Nevus (NEV)

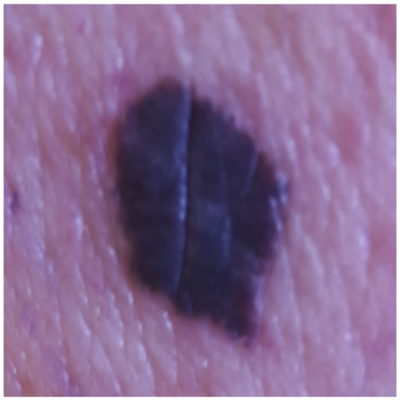

(f) Melanoma (MEL)

Figure S3: Diagnostic diversity on the PAD-UFES-20+ dataset. Representative smartphone-captured clinical images are shown for each of the six evaluated classes: (a) Actinic keratosis (ACK), (b) Basal cell carcinoma (BCC), (c) Squamous cell carcinoma (SCC), (d) Seborrheic keratosis (SEK), (e) Nevus (NEV), and (f) Melanoma (MEL).

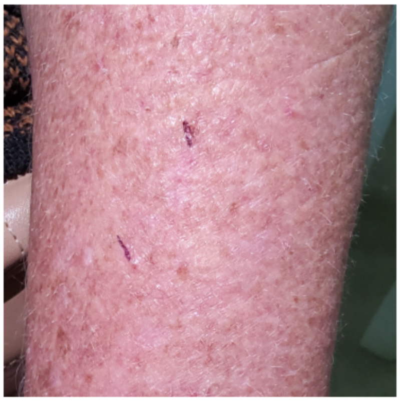

(a) Actinic Keratosis (ACK)

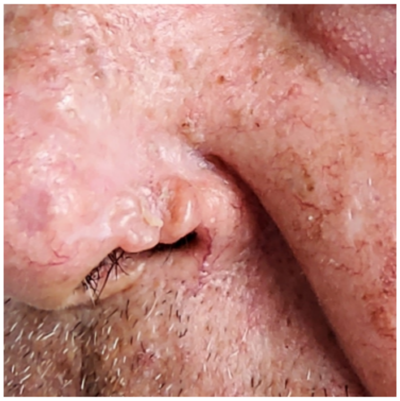

(b) Basal Cell Carcinoma (BCC)

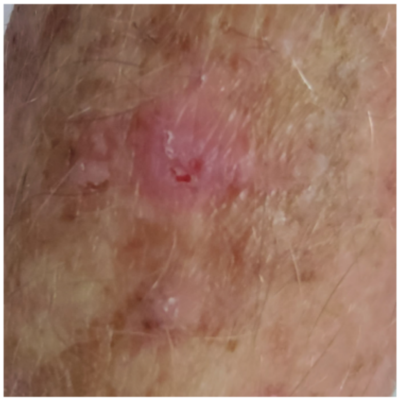

(c) Squamous Cell Carcinoma (SCC)

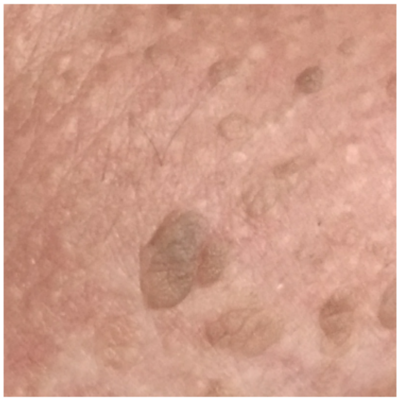

(d) Seborrheic Keratosis (SEK)

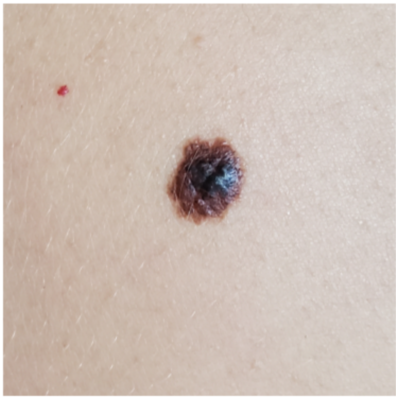

(e) Nevus (NEV)

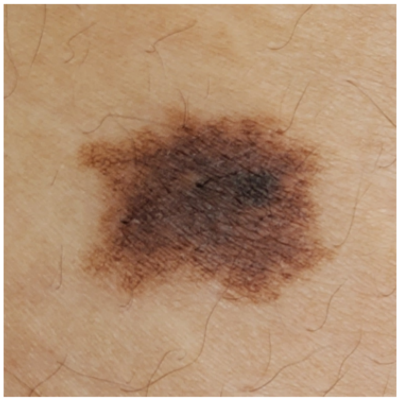

(f) Melanoma (MEL)

Figure S4: Diagnostic diversity on the MILK10k dataset[2]. Representative dermoscopic images are shown for each of the 11 evaluated classes: (a) Basal cell carcinoma, (b) Melanocytic nevus, (c) Benign keratinocytic lesion, (d) Squamous cell carcinoma/keratoacanthoma, (e) Melanoma, (f) Actinic keratosis/intraepidermal carcinoma, (g) Dermatofibroma, (h) Inflammatory and infectious, (i) Vascular lesions and hemorrhage, (j) Other benign proliferations, and (k) Other malignant proliferations.

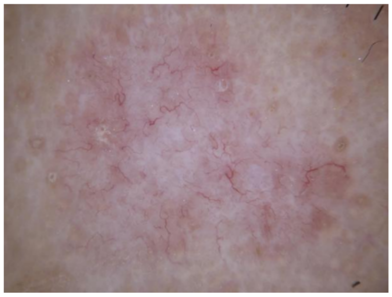

(a) Basal cell carcinoma

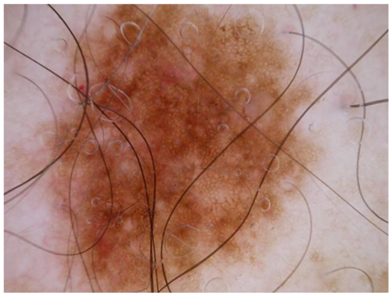

(b) Melanocytic nevus, any type

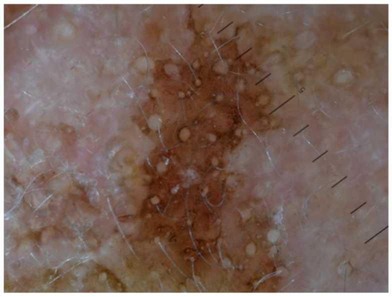

(c) Benign keratinocytic lesion

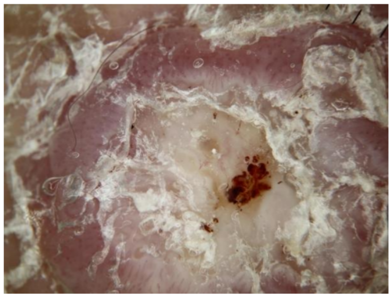

(d) Squamous cell carcinoma

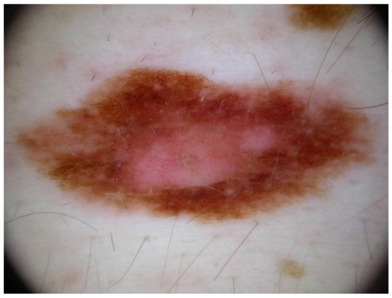

(e) Melanoma

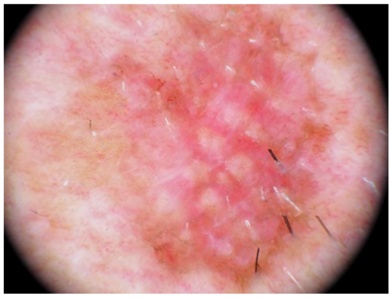

(f) Actinic keratosis

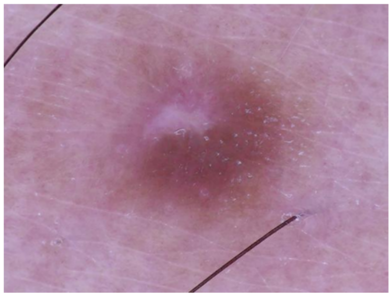

(g) Dermatofibroma

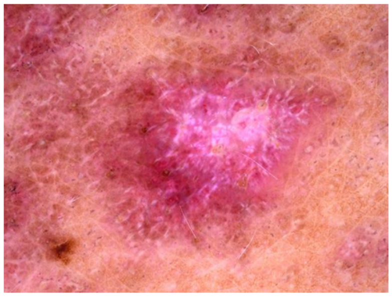

(h) Inflammatory/infectious

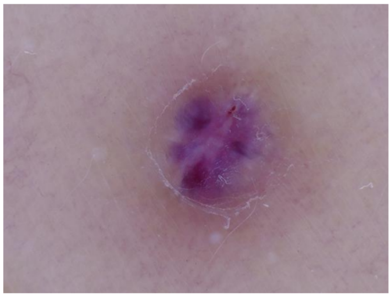

(i) Vascular lesions and hemorrhage

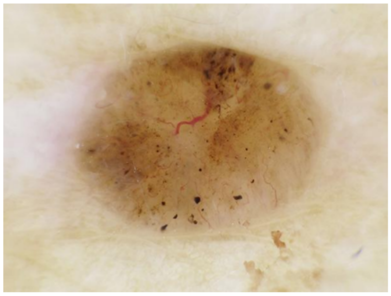

(j) Other benign proliferations including collisions

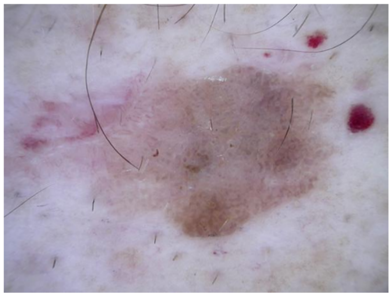

(k) Other malignant proliferations including collisions

Figure S5: Sensitivity analysis of stepwise validation performance on the PAD-UFES-20 under an alternative feature insertion order.

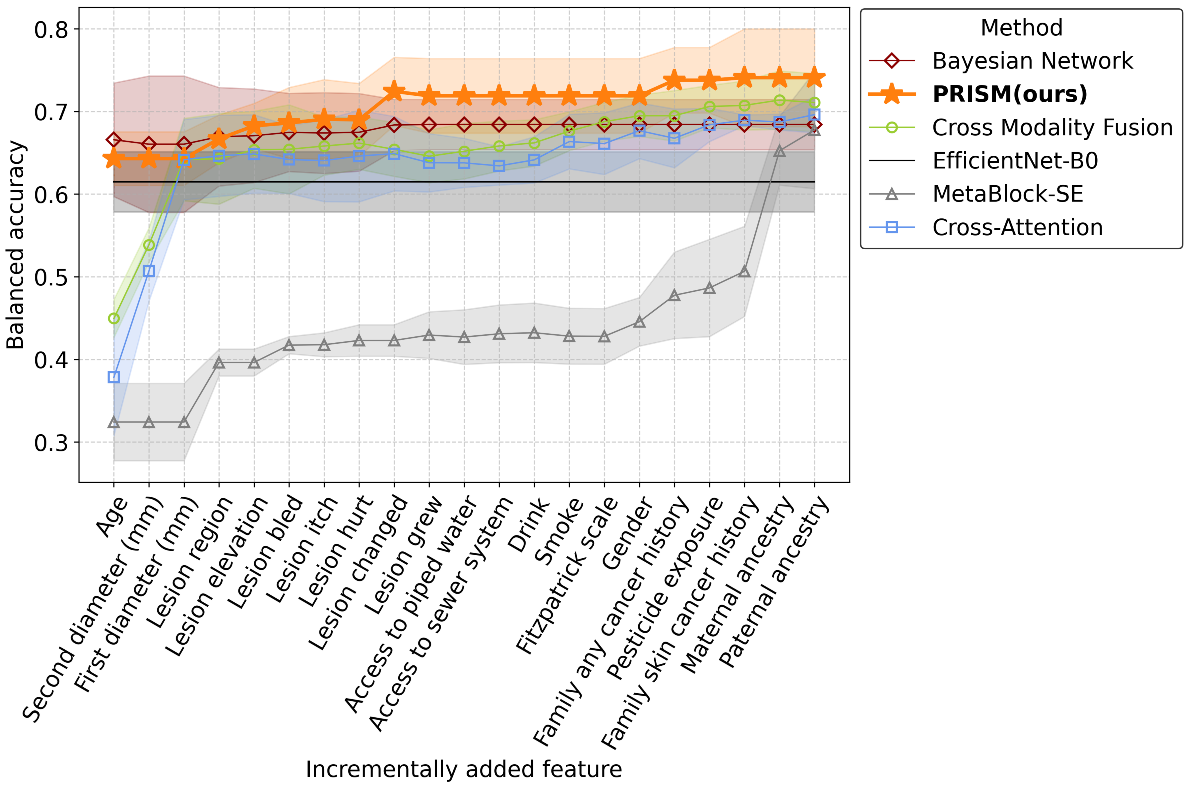

To assess the impact of feature sequence, patient metadata was incrementally introduced by first providing continuous numerical features (Age, Diameters), followed by categorical features ordered from least to most frequently missing. The proposed PRISM framework consistently maintains balanced accuracy above the vision-only EfficientNet-B0 baseline (solid black line) starting from the integration of the very first isolated feature. In contrast, end-to-end and attention-based fusion baselines exhibit severe early-step instability. When provided with an isolated, highly predictive feature (such as Age) amidst a vector of "missing" state indicators, these models suffer a precipitous drop in performance. They only recover and surpass the vision baseline after a critical mass of continuous are integrated. This behavior exposes a brittle architectural reliance on specific feature co-occurrences, whereas PRISM's conditional independence assumption provides safe integration of isolated clinical evidence. Shaded areas represent the 95% CI computed analytically using the Student's t-distribution.

Figure S6: Out-of-fold Confusion Matrix on the MILK10k dataset for the Siamese ResNet-50[3] baseline.

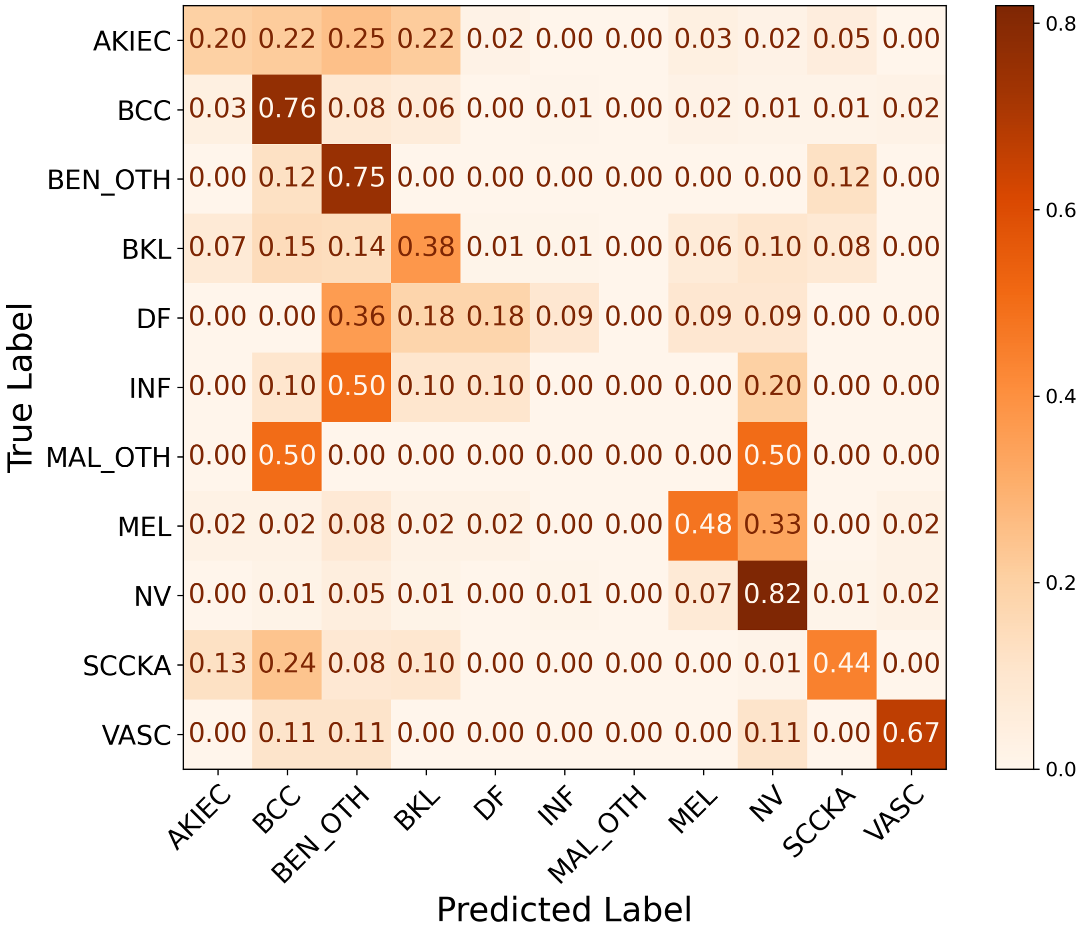

Both the baseline and our proposed method utilized a 5-fold cross-validation strategy stratified by target classes, and we ensured exact comparability by employing the same random seed to replicate the baseline's specific data splits.

Figure S7: Expected Calibration Error (ECE) reliability diagrams, across the EfficientNet-B0 and MobileNet-V3, on the PAD-UFES-20+ dataset.

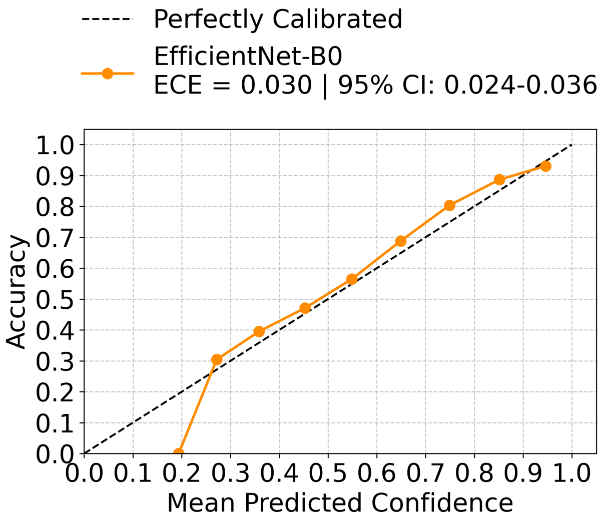

(a) EfficientNet-B0: Vision Baseline

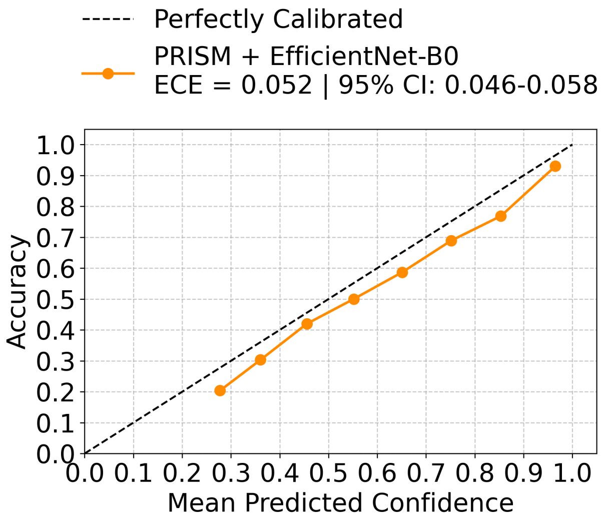

(b) EfficientNet-B0: PRISM

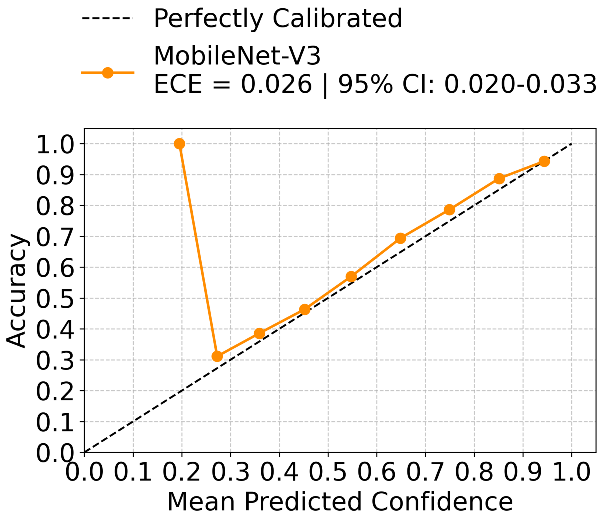

(c) MobileNet-V3: Vision Baseline

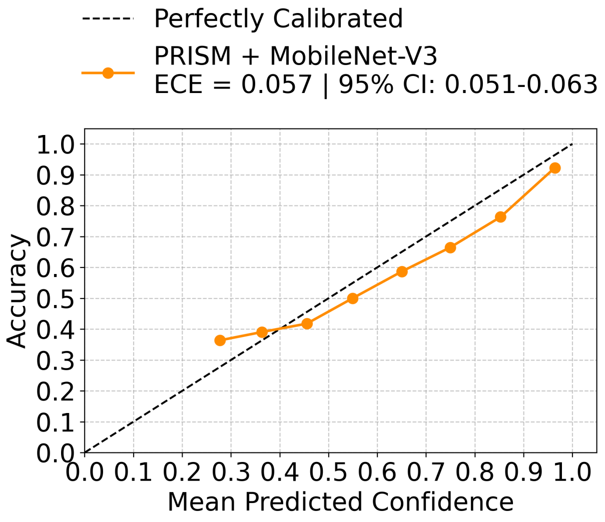

(d) MobileNet-V3: PRISM

Expected Calibration Error (ECE) reliability diagrams for the **PAD-UFES-20+** dataset. The left column illustrates the original vision-only baselines across the EfficientNet-B0 and MobileNet-V3 architectures. The right column demonstrates the confidence distributions after integrating clinical metadata via the uncalibrated PRISM framework, showcasing the baseline behavior of the fusion module.

Figure S8: Expected Calibration Error (ECE) reliability diagrams, across the Davit-Tiny and SwinV2-Tiny, on the PAD-UFES-20+ dataset.

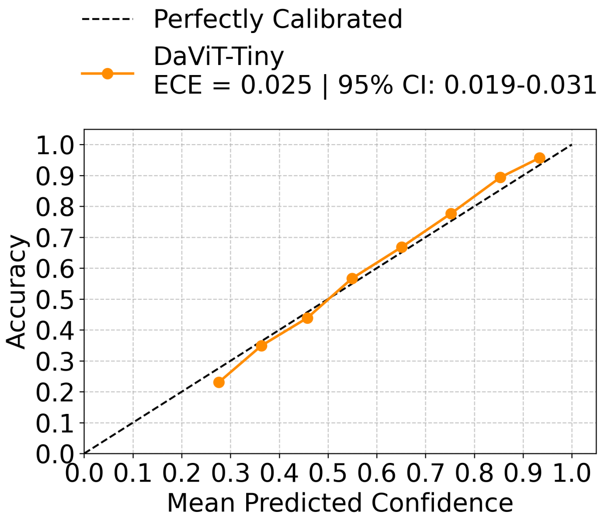

(a) DaViT-Tiny: Vision Baseline

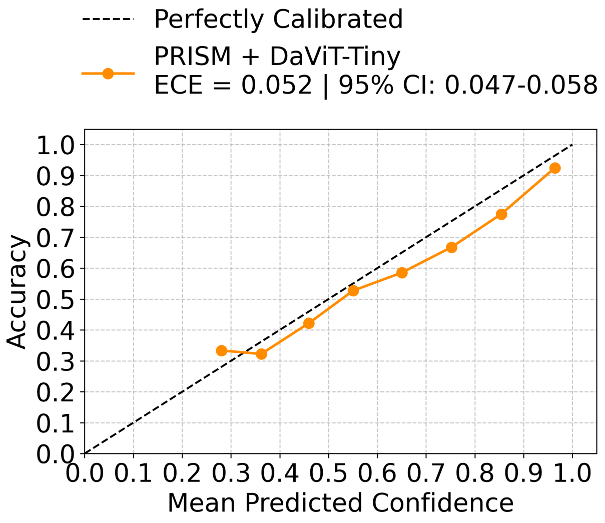

(b) DaViT-Tiny: PRISM

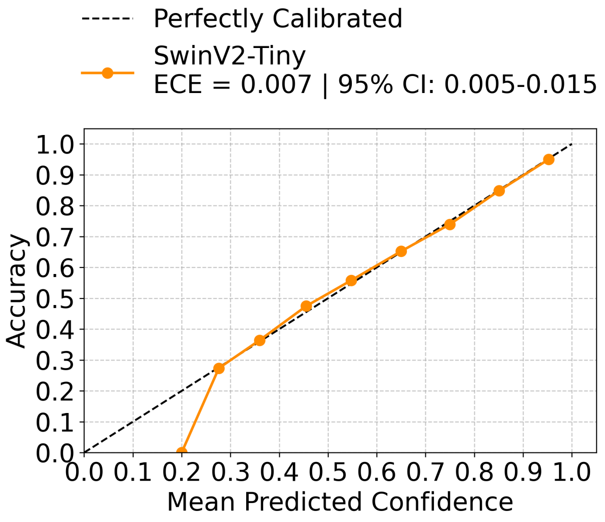

(c) SwinV2-Tiny: Vision Baseline

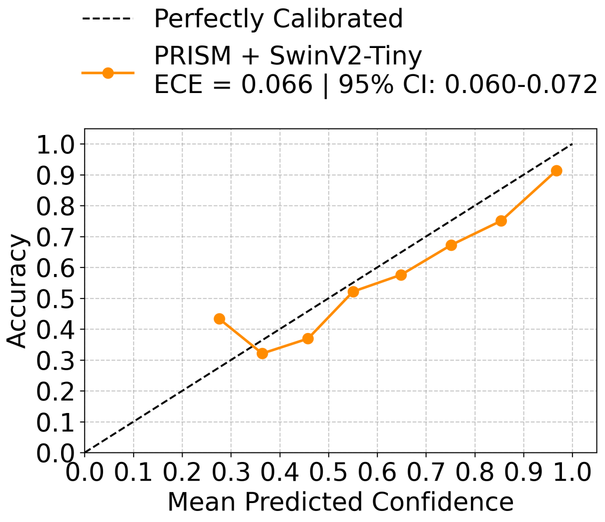

(d) SwinV2-Tiny: PRISM

Expected Calibration Error (ECE) reliability diagrams for the **PAD-UFES-20+** dataset. The left column illustrates the original vision-only baselines across the Davit-Tiny and SwinV2-Tiny architectures. The right column demonstrates the confidence distributions after integrating clinical metadata via the uncalibrated PRISM framework.

Figure S9: Expected Calibration Error (ECE) reliability diagrams, across the EfficientNet-B0 and MobileNet-V3, on the MILK10k dataset.

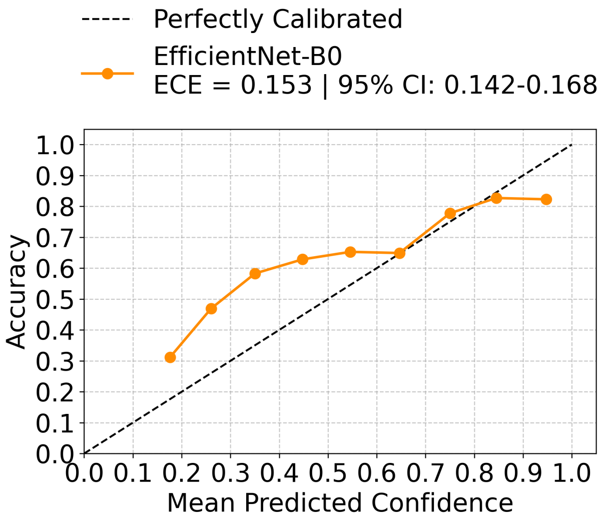

(a) EfficientNet-B0: Vision Baseline

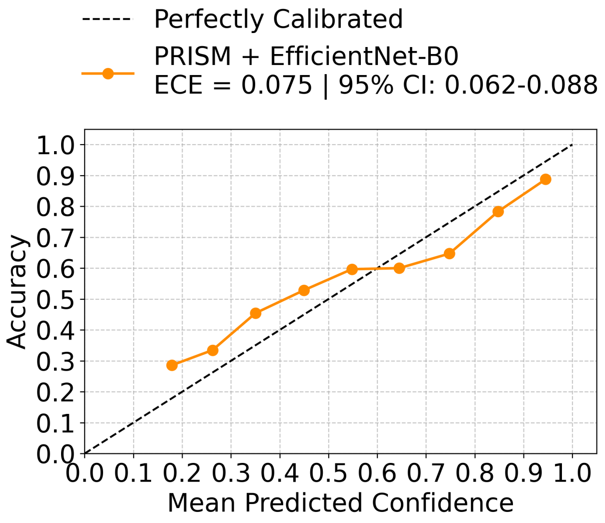

(b) EfficientNet-B0: PRISM

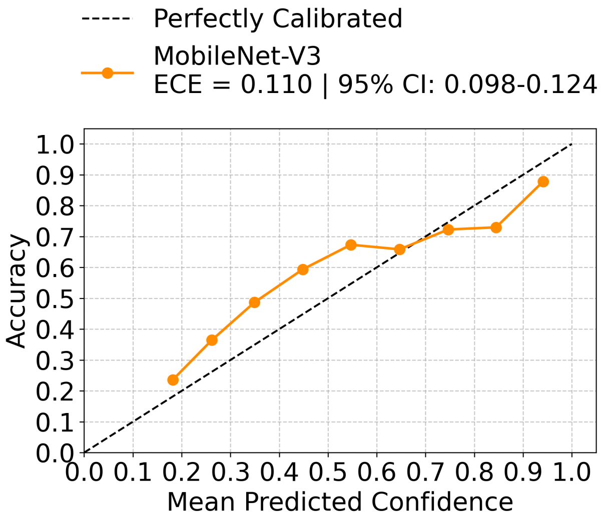

(c) MobileNet-V3: Vision Baseline

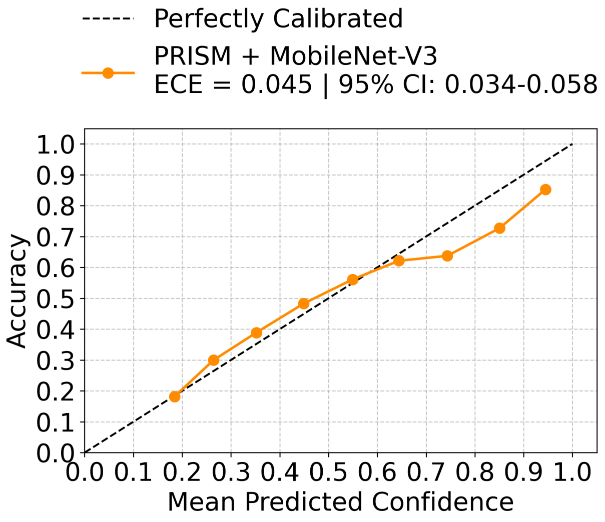

(d) MobileNet-V3: PRISM

Expected Calibration Error (ECE) reliability diagrams for the **MILK10k** dataset. The left column illustrates the original vision-only baselines across the EfficientNet-B0 and MobileNet-V3 architectures. The right column demonstrates the confidence distributions after integrating clinical metadata via the uncalibrated PRISM framework.

Figure S10: Expected Calibration Error (ECE) reliability diagrams, across the Davit-Tiny and SwinV2-Tiny, on the MILK10k dataset.

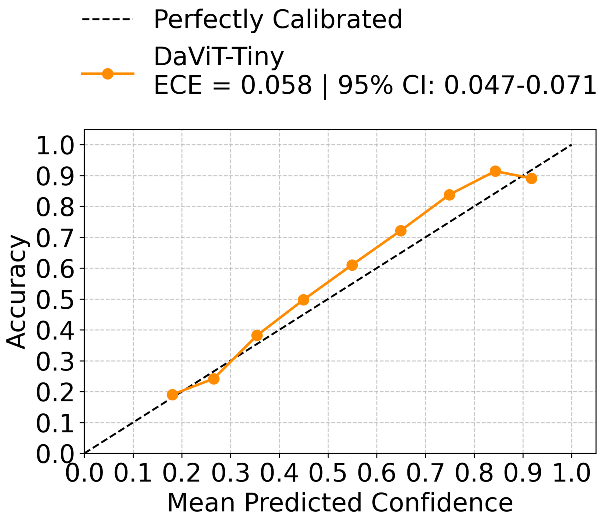

(a) DaViT-Tiny: Vision Baseline

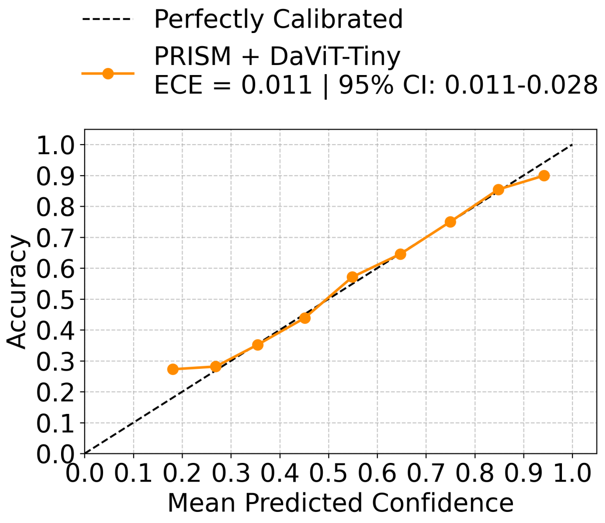

(b) DaViT-Tiny: PRISM

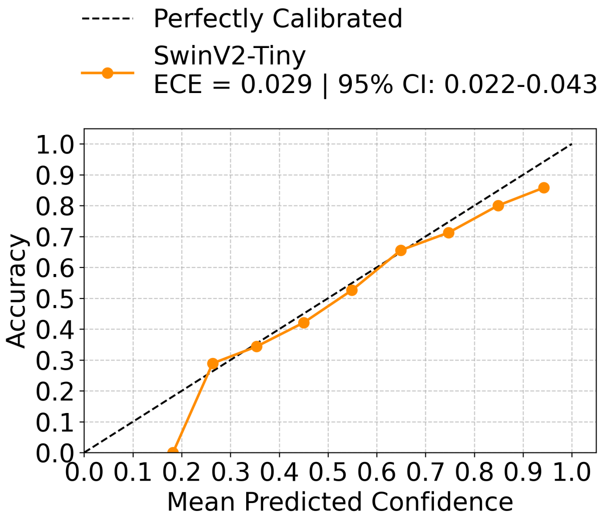

(c) SwinV2-Tiny: Vision Baseline

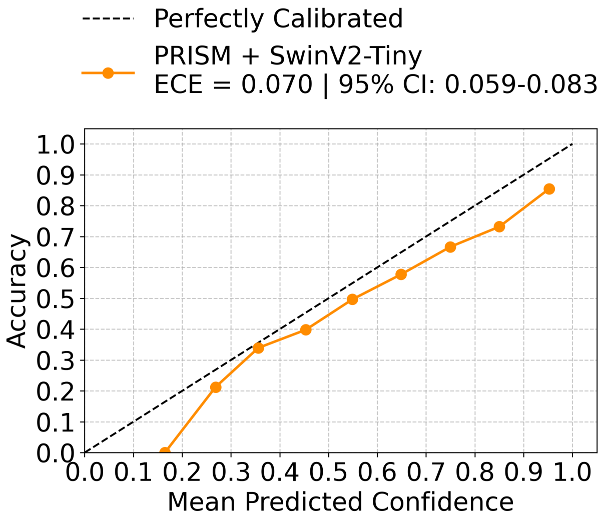

(d) SwinV2-Tiny: PRISM

Expected Calibration Error (ECE) reliability diagrams for the **MILK10k** dataset. The left column illustrates the original vision-only baselines across the Davit-Tiny and SwinV2-Tiny architectures. The right column demonstrates the confidence distributions after integrating clinical metadata via the uncalibrated PRISM framework.

Figure S11: Stepwise optimal temperature trajectory across backbones. The solid orange line represents the mean optimal temperature (T_N) across 5 cross-validation folds, with the shaded region denoting the 95% bootstrapped confidence interval (10,000 resamples). A clear monotonic increase in T_N as a function of the number of integrated metadata features (N) is observed for all architectures, empirically validating the compounding overconfidence inherent in the Naive Bayes formulation.

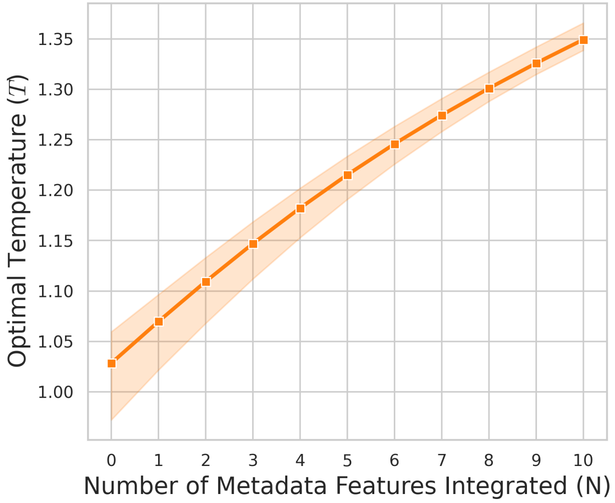

(a) DaViT-Tiny

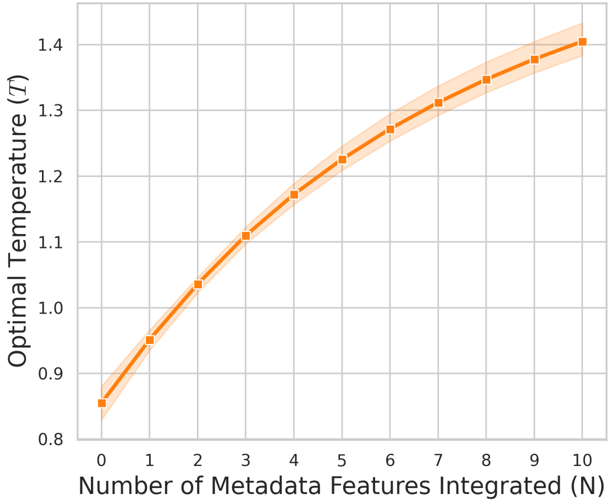

(b) EfficientNet-B0

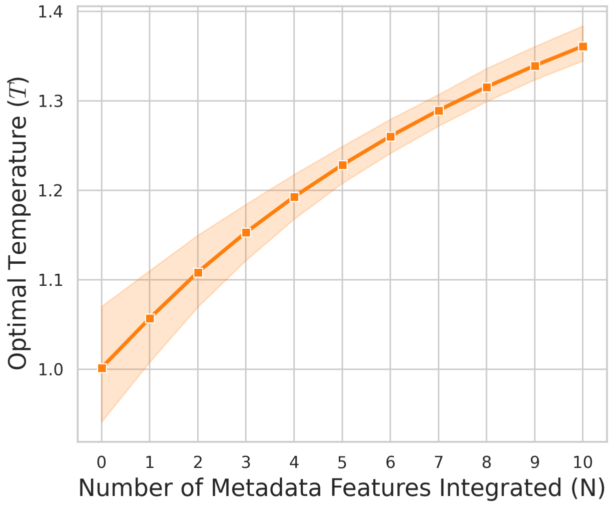

(c) MobileNet-V3

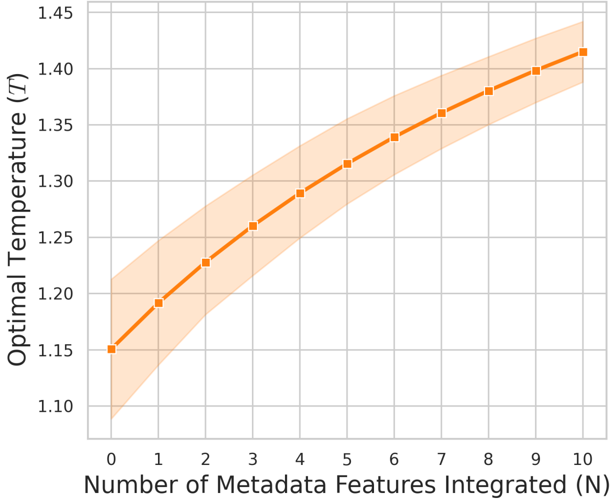

(d) SwinV2-Tiny

Figure S12: Inversion diagrams, across the evaluated backbones, on the PAD-UFES-20 dataset.

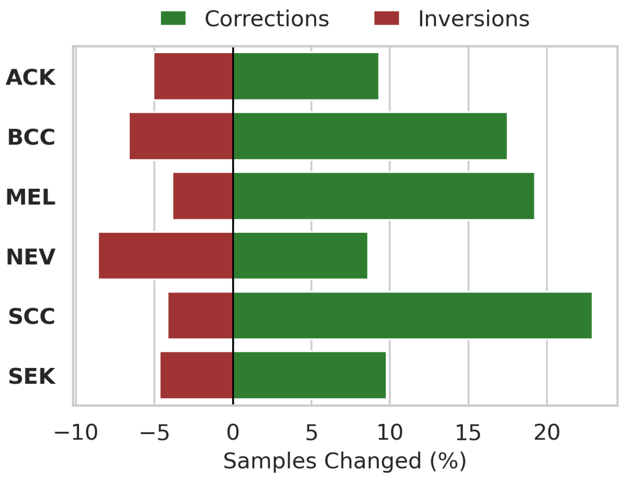

(a) DaViT-Tiny

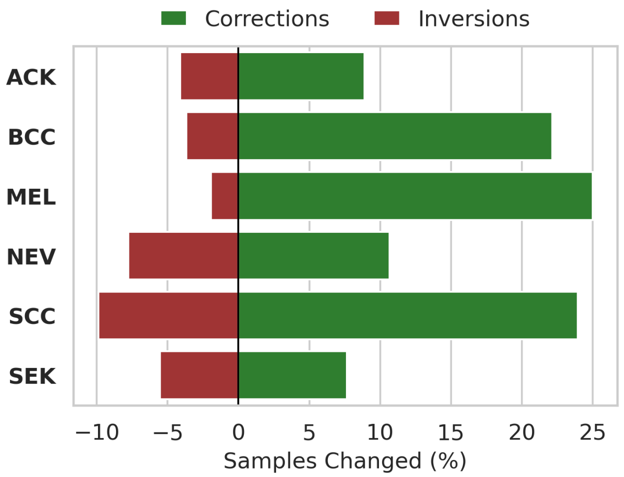

(b) SwinV2-Tiny

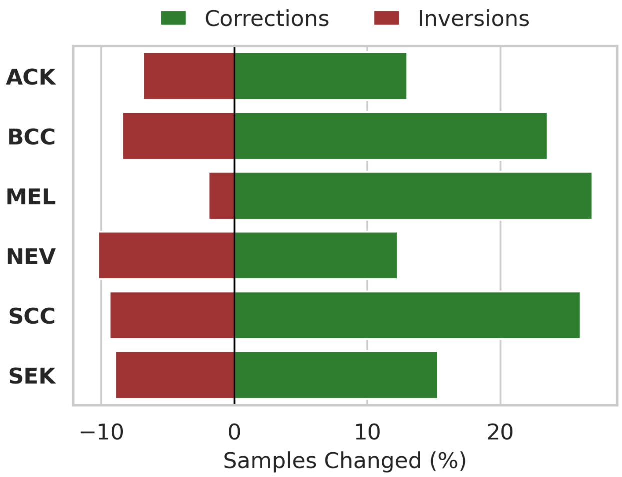

(c) EfficientNet-B0

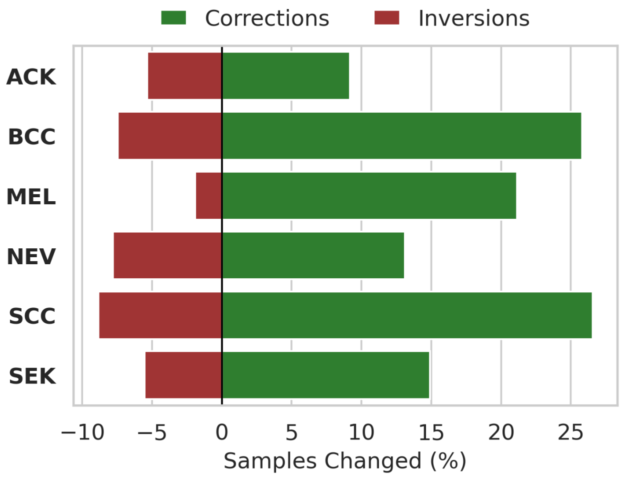

(d) MobileNet-V3

Per-class impact of PRISM clinical metadata integration across all four vision architectures. Green bars indicate the percentage of samples originally misclassified by the vision model that were successfully corrected by the metadata. Red bars represent originally correct vision predictions that were erroneously inverted. Results are pooled from out-of-fold validation.

Figure S13: Inversion diagrams, across the evaluated backbones, on the PAD-UFES-20+ dataset.

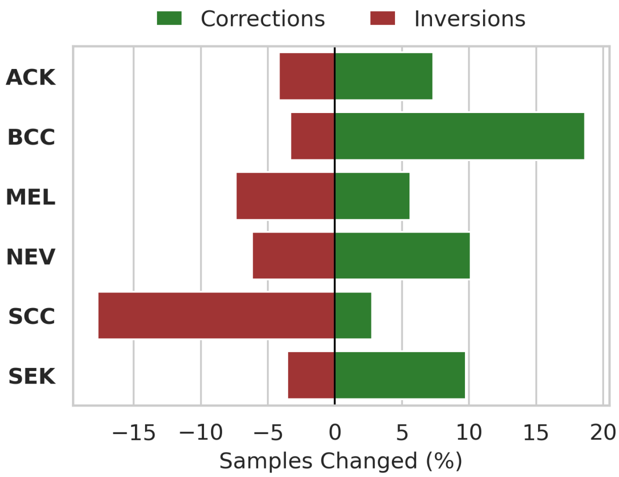

(a) DaViT-Tiny

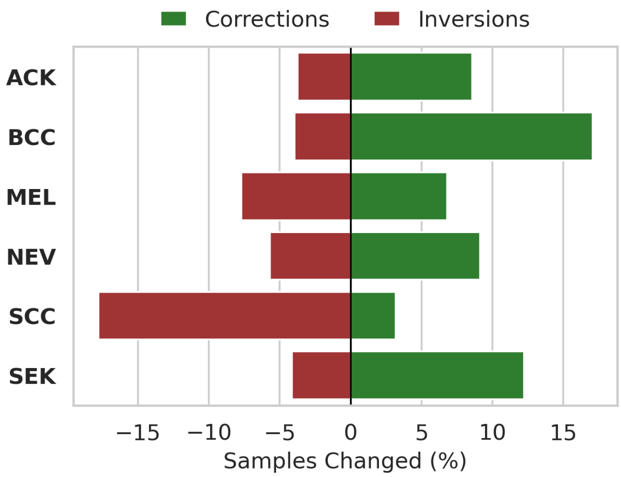

(b) SwinV2-Tiny

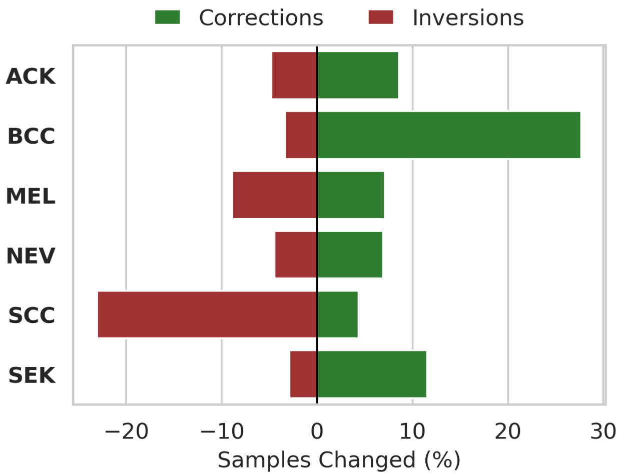

(c) EfficientNet-B0

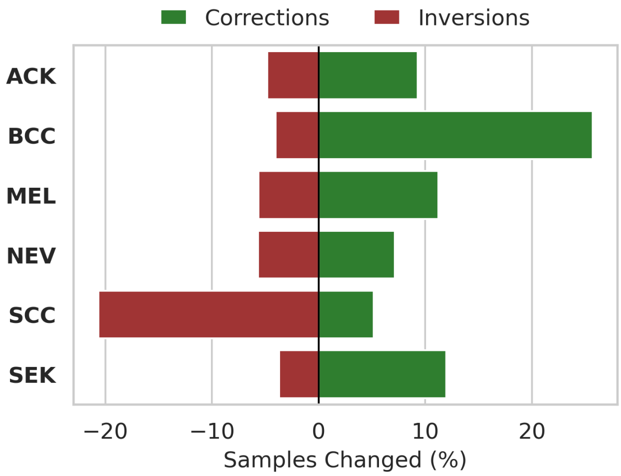

(d) MobileNet-V3

Per-class impact of PRISM clinical metadata integration across all four vision architectures. Green bars indicate the percentage of samples originally misclassified by the vision model that were successfully corrected by the metadata. Red bars represent originally correct vision predictions that were erroneously inverted. Results are pooled from out-of-fold validation.

Figure S14: Inversion diagrams, across the evaluated backbones, on the MILK10k dataset.

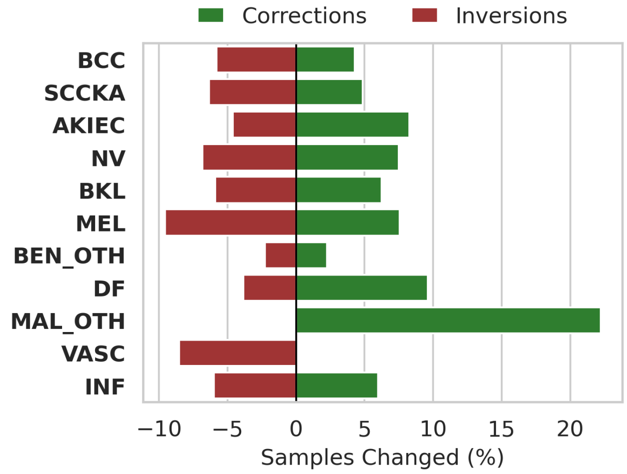

(a) DaViT-Tiny

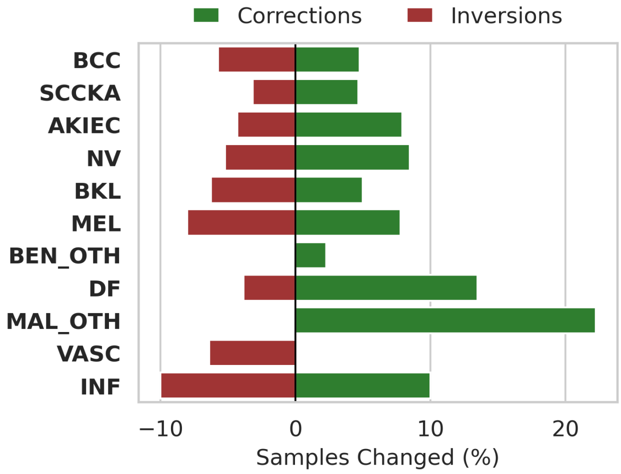

(b) SwinV2-Tiny

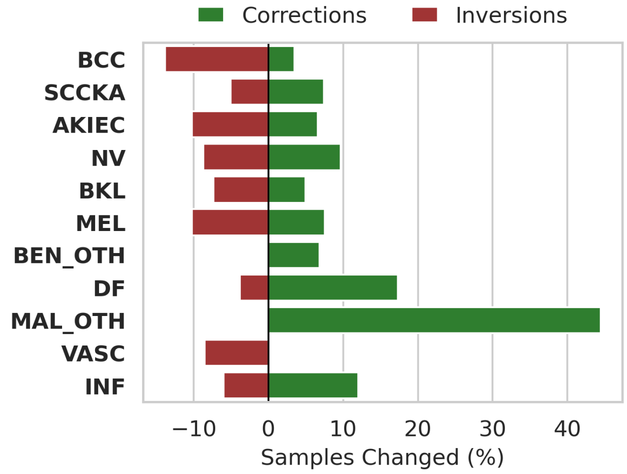

(c) EfficientNet-B0

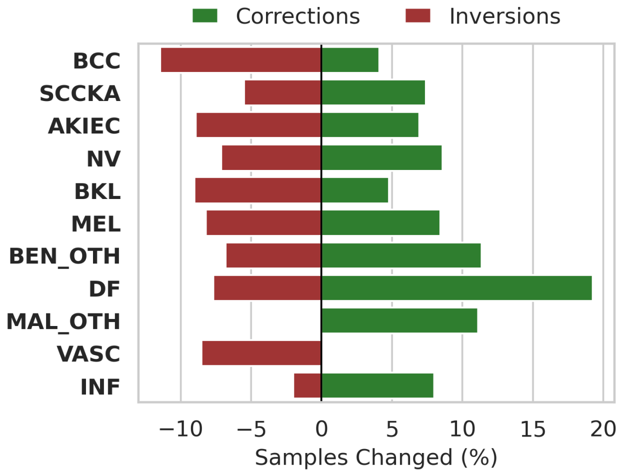

(d) MobileNet-V3

Per-class impact of PRISM clinical metadata integration across all four vision architectures. Green bars indicate the percentage of samples originally misclassified by the vision model that were successfully corrected by the metadata. Red bars represent originally correct vision predictions that were erroneously inverted. Results are pooled from out-of-fold validation.

Supplementary Tables

Table S1: Comparison of the number of samples for the PAD-UFES-20 and its extended version per class.

Label	PAD-UFES-20	PAD-UFES-20+
ACK	730	9190
BCC	845	3287
SCC	192	2371
SEK	235	727
NEV	244	405
MEL	52	338
**Total**	**2298**	**16318**

Table S2: Distribution of diagnostic classes in the MILK10k dataset.

Diagnostic class	No. of lesions	Percentage (%)
Basal cell carcinoma	2522	48.1
Melanocytic nevus, any type	746	14.2
Benign keratinocytic lesion	544	10.4
Squamous cell carcinoma/keratoacanthoma	473	9.0
Melanoma	450	8.6
Actinic keratosis/intraepidermal carcinoma	303	5.8
Dermatofibroma	52	1.0
Inflammatory and infectious	50	1.0
Vascular lesions and hemorrhage	47	0.9
Other benign proliferations including collisions	44	0.8
Other malignant proliferations including collisions	9	0.2

Table S3: Comparison of clinical features utilized by each fusion method on the PAD-UFES-20 dataset. A checkmark (✓) indicates the feature is explicitly utilized by the respective architecture.

Clinical Feature	PRISM	Cross-Attention	Cross Modality Fusion	MetaBlock-SE	Bayesian Network
Age	✓	✓	✓	✓	✓
Family skin cancer history	✓	✓	✓	✓	
Family any cancer history	✓	✓	✓	✓	
Lesion region	✓	✓	✓	✓	✓
Lesion grew	✓	✓	✓	✓	✓
Lesion itch	✓	✓	✓	✓	✓
Lesion bled	✓	✓	✓	✓	✓
Lesion hurt	✓	✓	✓	✓	✓
Lesion changed	✓	✓	✓	✓	✓
Lesion elevation	✓	✓	✓	✓	✓
First diameter (mm)		✓	✓	✓	✓
Second diameter (mm)		✓	✓	✓	✓
Gender		✓	✓	✓	
Drink		✓	✓	✓	
Smoke		✓	✓	✓	
Pesticide exposure		✓	✓	✓	
Fitzpatrick scale		✓	✓	✓	
Maternal ancestry		✓	✓	✓	
Paternal ancestry		✓	✓	✓	
Access to piped water		✓	✓	✓	
Access to sewer system		✓	✓	✓	

Table S4: Comparison of clinical features utilized by each fusion method on the PAD-UFES-20+ dataset. A checkmark (✓) indicates the feature is explicitly utilized by the respective architecture.

Clinical Feature	PRISM	Cross-Attention	Cross-Modality Fusion	MetaBlock-SE	Bayesian Network
Age	✓	✓	✓	✓	✓
Family skin cancer history	✓	✓	✓	✓	
Family any cancer history	✓	✓	✓	✓	
Lesion region	✓	✓	✓	✓	✓
Lesion grew	✓	✓	✓	✓	✓
Lesion itch	✓	✓	✓	✓	✓
Lesion bled	✓	✓	✓	✓	✓
Lesion hurt	✓	✓	✓	✓	✓
Lesion changed	✓	✓	✓	✓	✓
Lesion elevation	✓	✓	✓	✓	✓
Gender		✓	✓	✓	
Drink		✓	✓	✓	
Smoke		✓	✓	✓	
Pesticide exposure		✓	✓	✓	
Fitzpatrick scale		✓	✓	✓	
Maternal ancestry					
Paternal ancestry					
Access to piped water					
Access to sewer system					

Table S5: Comparison of clinical features utilized by each fusion method on the MILK10k dataset. A checkmark (✓) indicates the feature is explicitly utilized by the respective architecture.

Clinical Feature	PRISM	Cross-Attention	Cross-Modality Fusion	MetaBlock-SE	Bayesian Network
Age	✓	✓	✓	✓	✓
Gender	✓	✓	✓	✓	✓
Lesion region	✓	✓	✓	✓	✓
Skin tone	✓	✓	✓	✓	✓
Dermoscopic ulceration/crust		✓	✓	✓	
Dermoscopic hair		✓	✓	✓	
Dermoscopic vasculature/vessels		✓	✓	✓	
Dermoscopic erythema		✓	✓	✓	
Dermoscopic pigmented		✓	✓	✓	
Dermoscopic fluid/liquid		✓	✓	✓	
Dermoscopic skin markings		✓	✓	✓	
Clinical ulceration/crust					
Clinical hair					
Clinical vasculature/vessels					
Clinical erythema					
Clinical pigmented					
Clinical fluid/liquid					
Clinical skin markings					

Table S6: Global prediction transitions evaluated on the PAD-UFES-20 dataset. Values represent the percentage of pooled out-of-fold validation samples that were maintained correct, maintained wrong, corrected (vision-only error resolved), and inverted (vision-only correct prediction degraded) across the evaluated architectures following the integration of clinical metadata via the PRISM framework.

Backbone	Maintained Correct (%)	Maintained Wrong (%)	Corrections (+) (%)	Inversions (-) (%)	Net Improvement (%)
DaViT-Tiny	62.88	17.58	13.66	5.87	7.79
MobileNet-V3	55.53	19.84	18.02	6.61	11.40
EfficientNet-B0	54.96	18.49	18.45	8.09	10.36
SwinV2-Tiny	57.70	21.93	15.45	4.92	10.53

Table S7: Global prediction transitions evaluated on the PAD-UFES-20+ dataset. Values represent the percentage of pooled out-of-fold validation samples that were maintained correct, maintained wrong, corrected (vision-only error resolved), and inverted (vision-only correct prediction degraded) across the evaluated architectures following the integration of clinical metadata via the PRISM framework.

Backbone	Maintained Correct (%)	Maintained Wrong (%)	Corrections (+) (%)	Inversions (-) (%)	Net Improvement (%)
DaViT-Tiny	66.58	18.27	9.09	6.06	3.03
MobileNet-V3	59.46	21.50	12.10	6.94	5.17
EfficientNet-B0	60.47	20.50	11.88	7.15	4.73
SwinV2-Tiny	64.30	20.09	9.63	5.97	3.66

Table S8: Global prediction transitions evaluated on the MILK10k dataset. Values represent the percentage of pooled out-of-fold validation samples that were maintained correct, maintained wrong, corrected (vision-only error resolved), and inverted (vision-only correct prediction degraded) across the evaluated architectures following the integration of clinical metadata via the PRISM framework.

Backbone	Maintained Correct (%)	Maintained Wrong (%)	Corrections (+) (%)	Inversions (-) (%)	Net Improvement (%)
DaViT-Tiny	55.74	32.48	5.55	6.22	-0.67
MobileNet-V3	47.02	37.71	5.86	9.41	-3.55
EfficientNet-B0	50.15	33.42	5.67	10.76	-5.10
SwinV2-Tiny	50.94	37.65	5.84	5.57	0.27

References

1. Pacheco, A. G. *et al.* Pad-ufes-20: A skin lesion dataset composed of patient data and clinical images collected from smartphones. *Data Brief* **32**, 106221, DOI: https://doi.org/10.1016/j.dib.2020.106221 (2020).
2. MILK study team. MILK10k (2025).
3. Tschandl, P. *et al.* Milk10k: A hierarchical multimodal imaging-learning toolkit for diagnosing pigmented and nonpigmented skin cancer and its simulators. *J. Investig. Dermatol.* **146**, 357–364.e7, DOI: https://doi.org/10.1016/j.jid.2025.06.1594 (2026).
